# RNApedia: a database of structural protein–RNA interactions

**DOI:** 10.3389/fbinf.2026.1857218

**Published:** 2026-06-17

**Authors:** Luana Luiza Bastos, Diego Mariano, Pedro M. Martins, Rafael Pereira Lemos, Rafael Eduardo Oliveira Rocha, Leandro Morais de Oliveira, Sheila Cruz Araújo, Tatiane Senna Bialves, Raquel C. de Melo-Minardi

**Affiliations:** 1 Laboratory of Bioinformatics and Systems (LBS), Department of Computer Science, Universidade Federal de Minas Gerais, Belo Horizonte, Brazil; 2 Institute for Biochemistry and Molecular Biology, University of Hamburg, Hamburg, Germany

**Keywords:** curated dataset, machine learning dataset, molecular interfaces, PDB-derived data, protein-RNA interactions, structural bioinformatics

## Abstract

The interaction between RNAs and RNA-binding proteins (RBPs) is fundamental for gene expression and regulation of cellular homeostasis. The growing interest in understanding protein-RNA complexes and their use in developing biotechnological solutions has highlighted the need for computational resources to enable detailed structural analysis of these interactions. Despite the availability of structural databases, there is still a significant gap in specialized databases that integrate, in a curated, systematic, and up-to-date manner, structural information on these complexes. Here, we propose RNApedia, a specialized, curated database of protein-RNA complexes accessible via an interactive and user-friendly web interface. The database brings together systematic analyses of 56,133 protein-RNA pairs. It integrates structural descriptors, including accessible and hidden surface areas, atomic contacts and interaction types, RNA classification, protein domains, RNA modifications, and, when available, affinity data. RNApedia is a scalable and integrative platform for exploring protein-RNA interactions, serving as a promising resource for structural bioinformatics and data-driven approaches, including applications in artificial intelligence. All data are freely available for download at: https://bioinfo.dcc.ufmg.br/rnapedia.

## 1 Introduction

The interactions between RNA (ribonucleic acid) molecules and proteins, known as RNA-binding proteins (RBPs), are essential for maintaining cellular homeostasis and numerous regulatory processes (Poliseno et al., 2024; Mattick et al., 2023). Understanding the mechanisms governing these interactions within the protein-RNA complex is therefore essential for elucidating central aspects of molecular and cellular biology (Jones et al., 2001; Steinmetz et al., 2023). Alterations and defects in these interaction mechanisms are associated with various human pathologies, including cancer and neurodegenerative diseases, making these complexes relevant targets for the development of biotechnological solutions (Smith and Campbell, 2024; Ramanathan et al., 2019; Acharya et al., 2024).

Structural analysis of protein-RNA complexes has become increasingly relevant with the advancement of high-resolution experimental techniques such as cryo-electron microscopy. These approaches have enabled detailed three-dimensional analysis of RNA-protein interactions, including for large complexes such as ribosomal structures. However, the flexibility of RNA molecules, as well as the high cost and long time required to perform such analyses experimentally, remain major challenges in the study of RNA-protein complexes (Smith and Campbell, 2024).

Structural bioinformatics is a powerful auxiliary tool in the study of protein-RNA complexes, since it integrates experimental and computational data to model, analyze, and predict interactions of biological macromolecules. However, the application of this methodology faces significant challenges. In this context, we can highlight the limited availability of high-resolution structural data of protein-RNA in public repositories, the scarcity of specialized and regularly updated resources that offer comprehensive and easily searchable information on protein-RNA complexes, as well as the difficulty in dealing with the flexible nature of RNA. This lack of access to data restricts systematic studies, hinders the development of computational models focused on these interactions, and delays the advancement of rational RNA-based drug design strategies (Ramanathan et al., 2019; Acharya et al., 2024).

To address this challenge, we present RNApedia, a curated and continuously updated database of structural protein–RNA interactions designed to provide standardized, accessible, and user-friendly data for large-scale computational and artificial intelligence-driven analysis.

## 2 Materials and methods

**FIGURE 1:**
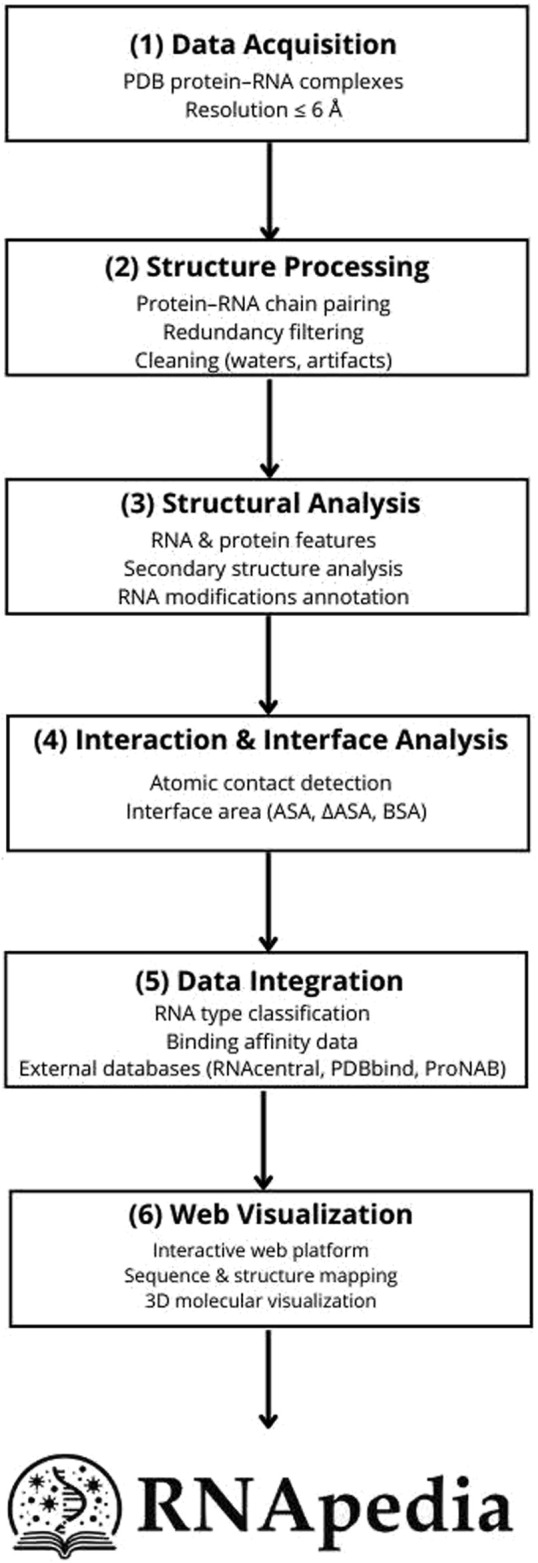
Overview of the RNApedia data processing, integration, and visualization pipeline. Protein-RNA complexes were obtained from the PDB and processed to generate interacting pairs, which were then analyzed structurally and interactionally. The resulting data were integrated with external resources for RNA classification and binding affinity annotation, and the results were finally presented through an interactive web platform with 3D and sequence-based molecular visualization.

Figure 1 summarizes the steps taken to collect the data that make up the database. Firstly, data were collected from the PDB database. We selected structures with at least one protein chain and one RNA chain, processed them, and separated protein-RNA pairs (distance ≤6 Å). We calculated a set of features using various approaches. Details will be presented below.

### 2.1 Data collection and processing

RNApedia was built using an automated pipeline to collect, filter, process, and annotate protein-RNA complexes deposited in the Protein Data Bank (PDB). PDB entries were retrieved directly from the RCSB PDB API (Burley et al., 2022), accessed on December 20, 2025, using custom Python 3 scripts.

Structures containing at least one protein and one RNA chain were selected. In addition, as a criterion for structure inclusion, structures obtained by X-ray crystallography, nuclear magnetic resonance (NMR), or cryo-electron microscopy with experimental resolution ≤6.0 Å were retained.

The structural files were processed to identify spatially close protein and RNA chains. The complexes were standardized into protein-RNA pairs to enable more detailed interface-level analyses and facilitate the processing of large, multi-stranded macromolecular structures. For each structure, protein-RNA pairs were initially defined based on the presence of at least one interatomic distance ≤6.0 Å between atoms belonging to the two chains. This criterion was used exclusively for interface neighborhood detection and pair generation, allowing the identification of spatially close protein-RNA chains. The selected threshold allows for the inclusion of potential intermolecular interaction neighborhoods, including long-range electrostatic interactions that can occur at distances close to 6.0 Å (Lemos et al., 2025). Similar conservative interface thresholds have also been adopted in other protein-RNA structural resources, including RNAproDB (Mitra et al., 2025), which uses a 6.0 Å atom-to-atom threshold for interface exploration and interaction neighborhood detection. The final attribution and classification of the interaction were subsequently refined using specific physicochemical and geometric criteria. Subsequently, a new structural file was generated for each identified protein-RNA pair. At the end of this stage, 88,435 protein-RNA pairs were generated.

Protein and RNA sequences were subsequently extracted from each protein-RNA pair for redundancy filtering. Sequence alignments were performed using BioPython 1.85 (Chapman and Chang, 2000), treating the protein-RNA pair as the unit of redundancy. Two pairs were considered redundant, and one was removed only when both the protein sequences and the corresponding RNA sequences simultaneously exhibited more than 95% sequence identity. This strategy was designed to eliminate nearly identical interaction pairs resulting from highly similar experimental structures, while preserving biologically relevant interaction diversity, including cases where the same protein interacts with different RNAs or the same RNA interacts with different proteins. After redundancy filtering, the final non-redundant dataset contained 61,051 protein-RNA pairs.

For standardization of structural data, non-structural water molecules, crystallographic artifacts, hydrogens, ligands, ions, and RNA modifications were processed separately. Water molecules were retained when they exhibited low thermal mobility and were spatially proximate to the protein-RNA interface. For this, we used the following criteria: a B-factor of less than 30.0 to search for more ordered and experimentally well-defined water molecules, since lower B-factor values generally indicate less conformational disorder and greater positional reliability (Barik and Bahadur, 2014; Spyrakis et al., 2017). Also, a maximum distance of ≤3.5 Å from any atom of the protein or RNA chain, a criterion compatible with typical distances associated with hydrogen bonds and frequently used to identify waters directly involved in structural stabilization and molecular recognition (Lemos et al., 2025). Crystallographic artifacts and hydrogen atoms were removed according to the criteria described in Supplementary Table S1 (Spyrakis et al., 2017). Small ligands were excluded from the structural files but retained as annotations in the database. Ions located up to 6.0 Å from the protein or RNA chain were retained and annotated. RNA modifications were identified using the MODOMICS database (2023 update) (Dunin-Horkawicz et al., 2006) and mapped to PDB residue identifiers using the RCSB PDB Ligand Expo feature (Westbrook et al., 2015). In total, 124 RNA modifications were identified, retained in the processed structures, and annotated in RNApedia (Supplementary Table S2).

For each protein-RNA pair, sequence-derived descriptors were calculated. For RNA molecules, sequence size, nucleotide composition, and GC content were determined using in-house scripts. For proteins, sequence size, molecular weight, aromaticity, instability index, and theoretical isoelectric point were calculated using the ProteinAnalysis class of Biopython version 1.85 (Chapman and Chang, 2000). Molecular weight was calculated from amino acid composition, considering the loss of one water molecule per peptide bond formed. Aromaticity was defined as the fraction of aromatic residues phenylalanine, tyrosine, and tryptophan in the sequence. The instability index was calculated according to the method of Guruprasad et al. (Guruprasad et al., 1990), and the theoretical isoelectric point was estimated as the pH at which the protein’s net charge is zero.

Structural annotation of RNA and protein components was also performed. RNA secondary structure information was obtained using both DSSR and RNAfold, which provide complementary structural perspectives. DSSR v2.5.4 (Lu et al., 2015) was used to identify canonical and non-canonical base pairs, stacking interactions, structural motifs (including hairpins, bulges, internal loops, and junctions), and secondary-structure annotations directly from experimentally resolved three-dimensional structures, reflecting conformations stabilized within the observed macromolecular complex. In contrast, RNAfold 2.5.1 from the ViennaRNA package (Lorenz et al., 2011) was used to predict RNA secondary structure based solely on sequence-derived thermodynamic models. Consequently, differences between both approaches are expected, particularly for RNAs whose conformations are influenced by protein binding, nucleotide modifications, tertiary interactions, or experimental conditions associated with structure determination. The combined use of DSSR and RNAfold provides a broader characterization of RNA structural properties in RNApedia. For proteins, STRIDE version 1.0 (Heinig and Frishman, 2004) was used to assign secondary-structure elements, while Pfam version 33.1 (Mistry et al., 2021; Bateman et al., 2004) was used to identify RNA-binding domains and motifs.

### 2.2 Contact analysis

Contact calculations were performed using a predefined methodology (Bastos et al., 2024). Interatomic contacts represent biochemical interactions between molecules and are computationally identified using geometric, distance, and physicochemical criteria. Distance-based approaches are among the most widely used methods for detecting intermolecular interactions (Silveira, 2008). Although interaction distance thresholds may vary depending on the type of interaction, these interactions are fundamental for stabilizing macromolecular structures and protein-RNA complexes (Silva et al., 2019; Alexandre et al., 2020).

To perform the contact calculations, the mapped RNA molecules and RNA modifications were processed using the Python RDKit library version 2025.03.5 (Landrum, 2025). For the four standard nucleotides, the LUNA library version 0.14.0 (Fassio et al., 2022) was used to predict atom types, classifying them as hydrogen bond donors, hydrogen bond acceptors, hydrophobic, aromatic, or charged.

Interactions were not classified solely based on distance thresholds. After identifying neighboring atoms, interactions were assigned based on their specific physicochemical compatibility and geometric constraints. The modified version of the COCαDA 1.0 script (Lemos et al., 2025; Lemos et al., 2024) was adapted to support the classification of nucleotide atoms and RNA modifications, and was used to analyze protein-RNA contacts.

Different interaction categories employed distinct distance and atom type criteria, including hydrogen bonds, hydrophobic interactions, attractive and repulsive electrostatic interactions, salt bridges, disulfide bonds, and aromatic stacking interactions. Additional geometric constraints were also applied to aromatic stacking interactions. The complete interaction classification criteria are summarized in Supplementary Table S3.

Protein-RNA pairs that did not exhibit any classified intermolecular interactions were removed from the database. At the end of this step, 56,133 protein-RNA pairs were retained in RNApedia.

### 2.3 Interface area analysis

Accessibility to the solvent and the interface area were evaluated using the ASA (Accessible Surface Area), ΔASA (Delta Accessible Surface Area), and BSA (Buried Surface Area) metrics. The ASA metric measures the area exposed to the solvent, while ΔASA represents the loss of accessibility after complex formation, represented by the equation:

$$\left(\Delta \text{ASA}=\text{ASA}\left(A\right)+\text{ASA}\left(B\right)-\text{ASA}\left(\text{AB}\right)\right)$$

BSA corresponds to the area buried at the interface, represented by the equation:

$$\left(\text{BSA}=½\times \left[\left(\text{ASA}\left(A\right)+\text{ASA}\left(B\right)\right)-\text{ASA}\left(\text{AB}\right)\right]\right)$$

The calculations were performed using NACCESS V2.1.1 (Ding and Arnold, 2006; Hubbard and Thornton, 1993). The analyses were performed considering total, polar, and nonpolar atoms, allowing the evaluation of the contributions of different physicochemical components to the interaction interface.

### 2.4 RNA type classification

The RNA molecules in RNApedia were classified using a three-step workflow that combined automated annotation and manual curation. First, RNAcentral (Petrov et al., 2017; The RNAcentral Consortium, 2019; The RNAcentral Consortium et al., 2015) was consulted via its API (accessed on December 20, 2025) using sequence-based identifiers, thereby directly annotating 13,863 of the 56,133 analyzed entries. Next, the remaining sequences were submitted to the Infernal (INFERence of RNA ALignment) tool (Nawrocki et al., 2009) (version 1.1.5) using covariance models from Rfam (Ontiveros-Palacios et al., 2025) version 15.1, which jointly consider sequence and secondary-structure conservation. In this step, 36,189 entries were classified. Finally, the remaining structures were submitted to manual curation by inspecting the corresponding PDB files and their associated publications. This procedure enabled the classification of 3,998 additional entries, leaving 2,083 cases unclassified.

### 2.5 PDBbind and ProNAB

Experimental protein-RNA binding affinity data were integrated into RNApedia from the databases PDBbind (2020 version) (Wang et al., 2005; Wang et al., 2004) and ProNAB (updated version, February 2023) (Harini et al., 2022). PDBbind (Wang et al., 2005; Wang et al., 2004) is a structural database of biomolecular complexes that compiles experimental affinity measurements, such as dissociation constants (Kd). From PDBbind (Wang et al., 2005; Wang et al., 2004), 244 protein-RNA complexes were associated with RNApedia, and their respective Kd values were collected. Since the number of protein-RNA complexes available in PDBbind was limited, additional data were obtained from ProNAB (Harini et al., 2022), a comprehensive database of protein-nucleic acid interactions that includes thermodynamic and experimental measurements. From ProNAB (Harini et al., 2022), 275 protein-RNA complexes associated with PDB identifiers were retrieved, from which dissociation constant (Kd) and binding free energy (ΔG) values were extracted.

### 2.6 Creating the web application

The RNApedia web application was developed using the CodeIgniter framework (v4.6.0) (https://codeigniter.com/), chosen for its lightweight architecture, integrated security features, and support for rapid development. Interactive tables were created with the DataTables library (v1.13.7) (https://datatables.net), while the user interface is structured with the Bootstrap framework (https://getbootstrap.com). Three-dimensional visualization of molecular structures is performed using 3Dmol.js (v2.5.4) (Harini et al., 2022), which allows interactive exploration of protein-RNA complexes directly in the browser.

## 3 Results and discussion

In this study, we present RNApedia, a database of protein-RNA interaction pairs. RNApedia was built to provide a comprehensive, curated resource for exploring the structural and functional aspects of protein-RNA interactions, supporting data-driven analyses, method development, and applications in structural bioinformatics and machine learning. RNApedia is available at https://bioinfo.dcc.ufmg.br/rnapedia.

**FIGURE 2:**
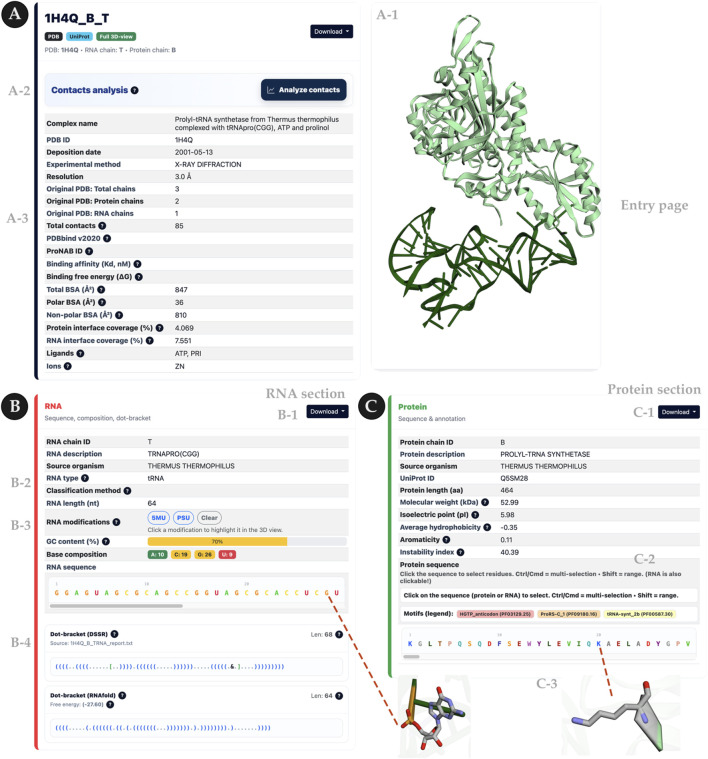
RNApedia web tool interface. **(A)** Overview of the analysis page for each entry of the protein-RNA complex in RNApedia. (A-1) 3D visualization of the protein-RNA complex. (A-2) Contacts analysis section. (A-3) Complex metadata panel, including PDB identification, experimental method, resolution, number of chains, total contacts, buried surface areas (BSA), interface coverage, presence of ligands and ions. **(B)** RNA structural information and analysis panel. The RNA report option (B-1) allows downloading a complete report containing RNA annotations and analyses. The RNA type and Classification method section (B-2) specifies the RNA’s functional class and the method used for its annotation. The RNA modifications area (B-3) highlights the chemical modifications present in the sequence. The lower Dot-bracket section (B-4) presents the RNA secondary structure in DSSR and RNAfold formats. **(C)** Information panel, functional annotation, and structural visualization of the protein in RNApedia. The Protein report option (C-1) allows you to download a complete protein report. The lower section, Protein sequence (C-2), displays the amino acid sequence interactively. Residue visualization (C-3).

In the RNApedia interface, each entry has an individual page with general information about the complex (Figure 2A), extracted data on the RNA chain (Figure 2B), and data on the protein chain (Figure 2C). The RNA chain panel presents detailed information on the selected RNA chain, including chain identification, functional description, organism of origin, RNA type, and classification method. On the other hand, the protein chain panel provides information about the protein’s structure, including structural and functional annotations, as well as the sequence. Each RNApedia entry is identified with a code of at least eight characters, the first four being the corresponding PDB-ID, a separator “_”, the code corresponding to the protein chain, another separator “_”, and finally, the code corresponding to the interacting RNA chain. For example, in the entry 1HCG_B_T (Prolyl-tRNA synthetase from *Thermus thermophilus* complexed with tRNApro), 1HCG corresponds to the PDB code, T corresponds to the RNA chain, and B corresponds to the protein chain (Figure 2). Further details about the interface can be found in Supplementary Text S1.

### 3.1 Data analyses

RNApedia currently contains 56,133 protein-RNA pairs, originating from 5,015 unique PDB files. Regarding experimental resolution methods, 49,384 complexes, corresponding to 88% of the structures present in the database, were resolved using cryo-electron microscopy (cryo-EM), while 6,591 (11.7%) were resolved by X-ray crystallography. In addition, there are a small number of structures resolved by other methods, among them Solution NMR, which corresponds to a small portion of the total set with 152 structures (0.3%), Solid-state NMR with five resolved structures (0.01%), and finally Fiber diffraction (six structures), historically used for the study of periodic arrangements and biological fibers. This result is expected, since cryo-EM is a technique that has evolved in recent years and facilitates the resolution of the structure of large macromolecular complexes, without the need for crystallization, as is the case with crystallography, and allows the resolution of larger complexes (Ma et al., 2022).

In Supplementary Table S4, we assess the database composition by RNA type. There is a predominance of ribosomal RNAs (rRNAs), representing 38,068 protein-RNA pairs (67.8%) in 1,248 unique PDB entries. This representation is consistent with the central role of ribosomes in cell biology and the growing interest in their study, particularly regarding targeted drugs. This characteristic also reflects the experimental efforts of recent decades to elucidate the structures of large complexes, such as ribosomes, at high resolution.

It is worth noting that, in RNApedia, the unit of analysis is the protein-RNA pair. This enables detailed characterization of interfaces and their properties within larger macromolecular complexes, facilitating their thorough evaluation. However, this approach also introduces a bias: ribosomal complexes, because they contain multiple protein and RNA chains in a single structure, generate a large number of protein-RNA combinations per PDB entry. Consequently, ribosomal RNAs (rRNAs), which already have a greater number of resolved structures compared to other RNAs complexed with proteins, are overrepresented compared to other types of RNA, which are generally associated with a smaller number of interactions per structure.

In addition to ribosomal RNAs (rRNAs), RNApedia presents a considerable diversity of RNA classes. For examples include RNAs classified as miscellaneous RNAs (miscRNA; 3,861 pairs; 6.9%; being 1,173 unique entries), synthetic RNAs (3,834 pairs; 6.8%; being 1,156 unique entries), as well as small nuclear RNAs (snRNAs; 2,459 pairs; 4.4%; 133 unique entries) and transfer RNAs (tRNAs; 2,423 pairs; 4.3%; being 712 unique entries), highlighting the heterogeneity of RNA types represented in the database.

Despite this diversity, some biologically relevant classes remain underrepresented in RNApedia, as well as in the PDB when evaluating protein-RNA pairs. Such as long non-coding RNAs (lncRNAs), microRNAs (miRNAs), and small interfering RNAs (siRNAs), which play essential roles in gene regulation, post-transcriptional control, and epigenetic processes. This underrepresentation is directly linked to the experimental challenges inherent in the structural determination of RNA molecules. In general, RNA exhibits high conformational flexibility, pronounced structural dynamics, and a strong dependence on environmental conditions for stabilizing its three-dimensional structure, making it difficult to obtain high-resolution structures using techniques such as X-ray crystallography and cryo-EM.

It is expected that, in future work, the number of available structures for these classes will increase, given their relevance to the study of protein-RNA interactions and their biotechnological and therapeutic applications. Furthermore, recent advances in structural modeling tools, such as AlphaFold3 (Elofsson, 2026), which can be used to predict protein-RNA complexes, have the potential to significantly expand the study of these interactions. In this context, future versions of RNApedia may incorporate predicted structural models of protein-RNA complexes, thereby expanding the database’s structural coverage and complementing the still-limited experimental data.

RNApedia integrates annotations of structural motifs at the protein interface, the presence of ions up to 6.0 Å from the protein-RNA pair, nucleotide modifications, and water molecules at a maximum distance of ≤3.5 Å from any atom of the protein or RNA chain, as well as experimental affinity information from the ProNAB and PDBbind databases (Table 1).

**TABLE 1:** Summary of structural and functional annotations present in RNApedia.

Feature	protein-RNA pairs(n)	Percentage (%)
PDBbind experimental binding affinity presence	650	1.22
ProNAB experimental binding affinity presence	538	1.01
Ions present	27,889	49.70
RNA modifications present	16,053	28.60
water molecules present	7,835	13.96
Pfam binding motifs present	52,466	98.77

The table presents the absolute numbers and percentages of RNApedia entries with experimental affinity data (PDBbind and ProNAB), the presence of ions, chemical modifications, and binding motifs (Pfam).

Regarding experimental affinity data, the database currently has very low coverage: only 1.22% of the structures have experimental affinity annotations from PDBbind (Wang et al., 2005; Wang et al., 2004) and 1.01% (538) from ProNAB (Wang et al., 2004). It is understood that this low coverage is due to the difficulty in obtaining this data in a free and accessible way. In future versions, it is intended to increase the number of structures with experimental affinities reported in the literature.

Analysis of the structural and functional annotations shows that approximately 49.7% of the pairs present ions near the protein-RNA interface (27,889 cases), highlighting their central role in stabilizing RNA structure and mediating interactions at the protein-RNA interface.

We observe chemical modifications in RNA nucleotides in 28.6% of protein-RNA pairs (16,053 cases), indicating that a significant fraction of RNA molecules undergo such alterations. These modifications can influence the stability, folding, and recognition of RNA by proteins, thereby modulating the specificity of interactions.

Potentially structural water molecules were identified in 13.96% of the structures (7,835 cases) using specific criteria to select potentially relevant water molecules at the protein-RNA interface. Structural water molecules play an important role in mediating indirect interactions, such as hydrogen bonds between proteins and RNA, and are fundamental to the stability and specificity of these interfaces.

In contrast, Pfam binding motifs are identified in 98.77% of protein-RNA pairs (52,466 cases), reflecting the wide availability of domain-level functional annotation for proteins. The near-total presence of Pfam domains provides a solid foundation for large-scale analyses and the development of computational approaches, including machine learning methods for studying protein-RNA interactions.

Supplementary Figure S1 presents the distribution of the main structural and functional components of protein-RNA interactions in RNApedia, comparing the complete dataset with that obtained after excluding ribosomal RNAs (rRNAs). In general, rRNA is observed to exert a dominant influence across all analyzed profiles, particularly on RNA modifications, Pfam domains, and ion distribution. In Supplementary Figure S1A,B, regarding RNA modifications, the complete set is strongly dominated by classic modifications associated with rRNAs. There is a strong predominance of classic structural modifications, such as pseudouridine (PSU) and different forms of methylation (5MC, 2 MG, OMG), reflecting the high degree of chemical modification characteristic of ribosomal RNAs, which perform highly conserved structural functions in the ribosome. These modifications are largely associated with the stabilization of RNA’s three-dimensional structure and the optimization of interactions with proteins and other molecules (Zhang et al., 2026; Li et al., 2025; Liu et al., 2024; Liu and Pan, 2016).

In contrast, when excluding rRNAs, not only is there a significant reduction in the absolute frequency of these modifications, but also a change in the relative profile, with an increase in the diversity of modified types. In this scenario, modifications more frequently associated with functional RNAs, such as tRNAs and regulatory RNAs, emerge, which are involved in dynamic processes, including gene expression regulation, molecular recognition, and functional adaptation.

The analysis of Pfam domains clearly shows how the dataset composition affects the interpretation of protein-RNA interactions. When all pairs are considered, Supplementary Figure S1C, an almost absolute predominance of ribosomal domains is observed, such as Ribosomal_L16, Ribosomal_L5, and Ribosomal_L30, reflecting the large number of ribosomal structures available in the PDB. These domains are associated with highly conserved structural proteins, whose main function is ribosome stabilization and coordination of the translation process. As a consequence, the overall domain profile is strongly biased towards structural and highly specialized functions, limiting the observed functional diversity (Lodde et al., 2023; Lunde et al., 2007).

However, by excluding ribosomal RNAs in Supplementary Figure S1D, a substantially different and more representative scenario of the functional diversity of protein-RNA interactions emerges. In this context, domains such as RRM_1 (RNA Recognition Motif) stand out, widely recognized as the main RNA-binding module in regulatory proteins and involved in processes such as splicing, transport, and RNA stability. The significant presence of LSM (Like-Sm) type domains reinforces the importance of ribonucleoprotein complexes involved in dynamic RNA metabolism processes, including maturation, splicing, and degradation, particularly in non-ribosomal contexts (Lodde et al., 2023; Lunde et al., 2007).

Additionally, the identification of domains associated with RNA polymerase, such as RNA_pol_Rpb1 and RNA_pol_Rpb2, indicates the representation of complexes directly involved in transcription, suggesting that the base also captures protein-RNA interactions in dynamic and functional contexts, in addition to static structures. The presence of RAMP domains, associated with CRISPR systems, further highlights the inclusion of molecular defense mechanisms and specific RNA recognition, expanding the biological spectrum covered by RNApedia (Auweter et al., 2006; Cieniková et al., 2015; Cléry et al., 2008).

In the Supplementary Figures S1E and S1F, the distribution of ions is shown; magnesium (Mg^2+^) is clearly dominant in both sets, highlighting its fundamental role in the structural stabilization of RNA. However, the removal of rRNAs S1 E significantly reduces their absolute frequency and increases the relative relevance of other ions, such as Zn^2+^, which are frequently associated with catalytic and structural functions in proteins (Draper, 2008; Reymond et al., 2009).

#### 3.1.1 Interface and atomic contact analysis

When evaluating the distribution of buried surface area (BSA) in protein-RNA pairs from RNApedia, an asymmetric profile is observed, with a predominance of small to medium interfaces and a long tail corresponding to more extensive interfaces (Supplementary Figure S2A). This behavior is consistent with classical structural analyses of protein-RNA interfaces, which show that, on average, these interactions tend to involve relatively compact surfaces, although some complexes, especially the larger ribonucleoproteins exhibit substantially larger interfaces. The maintenance of this profile even after the exclusion of rRNAs (Supplementary Figure S2B) indicates that this asymmetry does not only result from the overrepresentation of ribosomal complexes, but reflects a general property of protein-RNA recognition. In biological terms, this suggests that specificity and stability can emerge from highly optimized local arrangements, without the need for extremely extensive interfaces, which is particularly relevant for understanding transient regulatory interactions and for the rational design of molecules capable of modulating these interfaces (Bahadur et al., 2008).

The composition of the buried interface reveals, on the other hand, a marked predominance of nonpolar contributions, while polar fractions represent only about 6%–7% of the total area, both in the complete set and after the exclusion of rRNAs. At first glance, this result seems to contrast with the classical view that protein-RNA interactions are dominated by hydrogen bonds and electrostatic forces. However, this apparent contradiction can be resolved by a model in which electrostatic interactions guide initial recognition and molecular complementarity, while hydrophobic packing and solvent exclusion provide an important contribution to the thermodynamic stabilization of the complex. This point is particularly important for computational applications because it indicates that purely electrostatic descriptors are insufficient to represent the physics of these interfaces; more robust predictive models must simultaneously integrate geometry, atomic composition, polarity, solvent accessibility, and structural context (Bahadur et al., 2008; Jones et al., 2001).

**FIGURE 3:**
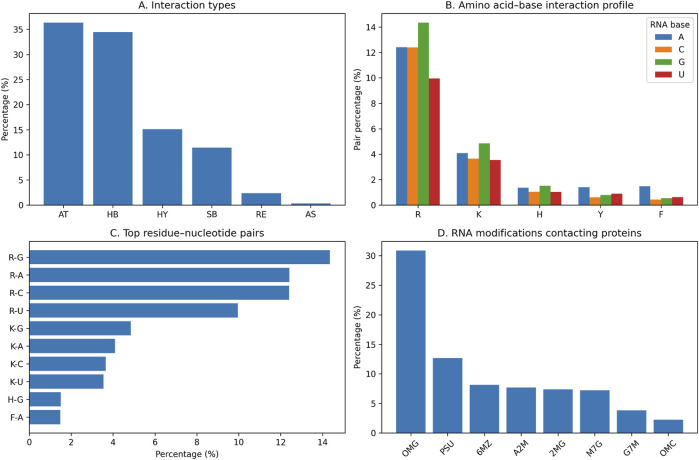
Overview of protein-RNA interaction patterns. **(A)** Distribution of interaction types observed at protein–RNA interfaces. **(B)** Amino acid-base interaction profile showing the contribution of the most frequent amino acids across RNA bases. **(C)** Most frequent residue-nucleotide interaction pairs, highlighting dominant interaction patterns. **(D)** RNA modifications contact proteins, highlighting the most frequently modified nucleotides at interaction interfaces.

This interpretation is consistent with the distribution of contact types observed in Figure 3A, in which attractive interactions (AT), hydrogen bonds (HB), and salt bridges (SB) predominate. This pattern reinforces the central role of electrostatic complementarity between the negatively charged phosphate backbone of RNA and positively charged protein residues. At the same time, the presence of hydrophobic interactions and aromatic stacking, although less frequent, shows that the protein-RNA interface cannot be reduced to an exclusively electrostatic model. In functional terms, this is relevant because the combination of directional contacts and packaging contributions helps explain how these interfaces reconcile molecular specificity with conformational plasticity. This mixed architecture is also highly informative for docking, scoring, and machine learning strategies, in which the correct weighting between different interaction types is crucial for predicting binding sites, affinity, and recognition modes (Bahadur et al., 2008; Jones et al., 2001; Steinmetz et al., 2023; Barik et al., 2015).

The comparison between the overall assemblies, rRNA complexes, non-rRNA complexes, and contacts involving modified nucleotides, presented in Supplementary Table S5, further clarifies this interpretation. While the overall assemblies and non-rRNA complexes exhibit similar profiles, dominated by electrostatic interactions and hydrogen bonds, the rRNA-containing complexes show a relatively greater contribution of hydrophobic interactions, likely reflecting the greater structural packing characteristic of large ribonucleoprotein assemblies. In contrast, contacts involving modified nucleotides show a relative increase in repulsive interactions, suggesting that RNA modifications may remodel the electrostatic microenvironment of the interface. This finding is consistent with the literature, indicating that RNA modifications not only alter local structural properties but also modulate how proteins recognize, stabilize, or discriminate specific RNA regions. From an applied perspective, this reinforces the importance of incorporating information about modifications into structural models and drug discovery pipelines, especially when the goal is to capture fine selectivity or regulatory mechanisms dependent on the molecular context (Lewis et al., 2017).

At the residue level, Figure 3B shows that positively charged residues, especially arginine, dominate interactions with different nitrogenous bases, while Figure 3C shows that residue-nucleotide pairs containing arginine are among the most frequent at the interface. This pattern is consistent with previous structural studies, which describe the central role of arginine and lysine in nucleic acid recognition, due to the ability of these residues to combine favorable electrostatic interactions with multiple hydrogen bonds. The fact that the distribution is relatively homogeneous among different bases suggests that, in many cases, specificity does not depend exclusively on nucleotide identity, but on physicochemical complementarity and the local geometry of the interface. This observation has important implications for structural biology and AI: instead of relying solely on sequence, predictive models of protein-RNA interaction need to capture spatial patterns, local charges, solvent exposure, and the three-dimensional organization of the interface (Bahadur et al., 2008).

The analysis of RNA modifications in direct contact with proteins, presented in Figure 3D, adds an additional layer of functional complexity. The fact that only a specific subset of modified nucleotides recurrently appears at the interface suggests that these modifications are not randomly distributed, but may contribute to the fine-tuning of local physicochemical properties, influencing charge distribution, geometry, hydration, and stability. This aspect is especially relevant in the current context of RNA-based therapies and the development of compounds that modulate RNA-protein interactions. Since modifications can alter affinity, recognition, and conformational dynamics, their explicit consideration can improve both the mechanistic interpretation of known complexes and the construction of more realistic predictive models for prioritizing therapeutic targets and designing molecules with greater selectivity (Lewis et al., 2017).

Taken together, the results shown in Figures 3A–D, Supplementary Figures S2A,B, and Supplementary Table S5 support a multifactorial model of protein-RNA recognition, in which electrostatic interactions and hydrogen bonds guide initial recognition and specificity, while hydrophobic contributions and solvent exclusion play a decisive role in stabilizing the complex. The additional modulation promoted by RNA modifications further amplifies this complexity and suggests a structurally relevant regulatory layer. From an applied perspective, this overview reinforces the value of integrative databases like RNApedia for three main areas: the mechanistic interpretation of protein-RNA interfaces, the development of machine learning models for predicting binding sites, contacts, and affinity, and the identification of structural principles useful for advancing RNA-targeted therapies or RNA-protein interactions (Wei et al., 2022).

### 3.2 Utility

RNApedia was designed as an accessible and easy-to-use resource for exploring protein-RNA structural data. By organizing structures into protein-RNA pairs, the database provides a comprehensive set of structural annotations, both sequence-based and interface-level, enabling detailed and large-scale analyses of the physicochemical and structural properties of these complexes. A key application of RNApedia is comparative analysis, enabling the systematic investigation of structural and sequence patterns across various protein-RNA interactions.

In recent years, advances in understanding different classes of RNA, including messenger RNAs (mRNAs) and non-coding RNAs (ncRNAs), have significantly expanded therapeutic possibilities (Sparber et al., 2019). Non-coding RNAs have been explored as therapeutic targets in various diseases, including cancer and cardiovascular pathologies (Lieberman, 2018; Stephenson and Zamecnik, 1978; Zamecnik and Stephenson, 1978). In parallel, different RNA-based therapeutic approaches are being developed, such as antisense oligonucleotides (ASOs) (McDowall et al., 2024; Khuu et al., 2024; Lauffer et al., 2024; Zhang, 2024), siRNAs (Xiong et al., 2022), and RNA vaccines (Abbasi, 2020; Miao et al., 2021), with molecules approved by international regulatory agencies. Among the most promising applications are aptamers, single-stranded oligonucleotides with high affinity and specificity for molecular targets, mainly proteins, obtained through techniques such as SELEX (Fang et al., 2024). These compounds show great potential in diagnostics, biosensors, and targeted therapies due to their low immunogenicity and high biocompatibility (Niazi et al., 2024; Yan et al., 2024). In this context, *in silico* approaches, combined with structural bioinformatics, play a fundamental role in the optimization of these molecules, a scenario in which RNApedia stands out as an essential platform, providing curated and organized structural data that enable the efficient application of *in silico* approaches, directly contributing to the optimization and rational development of these molecules.

In parallel, the growing application of Artificial Intelligence (AI) has transformed the study of protein-RNA interactions. Machine learning and deep learning methods have been employed to predict binding sites, identify interface residues, and characterize molecular affinities (Koike and Takagi, 2004; Šikić et al., 2009; Park et al., 2009). These approaches have evolved from models based exclusively on sequences to more sophisticated architectures, such as convolutional neural networks (CNNs), recurrent neural networks (RNNs), and Graph Neural Networks (GNNs), which integrate structural, sequential, and topological information. In addition, models based on biological language, such as protein and RNA embeddings, have expanded the generalization capacity of predictive systems (Elofsson, 2026; Wei et al., 2022; Koike and Takagi, 2004; Šikić et al., 2009; Park et al., 2009).

However, the performance of these models critically depends on the quality and standardization of the data used. Structural data available in public databases often present inconsistencies, heterogeneity, and annotation gaps, which can compromise the robustness of the models. In this context, RNApedia presents itself as a strategic tool, offering curated, standardized, and systematically organized data. The availability of selected datasets, customizable subsets, and files ready for use in computational pipelines enables the construction of training, validation, and benchmark sets for AI models.

In this way, RNApedia not only facilitates large-scale structural and comparative analyses but also drives the development of advanced computational approaches, thereby identifying recurring patterns in protein-RNA interfaces and advancing precision medicine. Its integration with artificial intelligence methodologies positions the platform as a central resource in the discovery and optimization of RNA-based therapies, consolidating its role at the interface between structural biology, bioinformatics, and biomedical innovation.

In the following subsections, we will present some examples of RNApedia use cases.

#### 3.2.1 Use case I: exploring proteins that take on RNA roles

Ribosomal RNA (rRNA) comprises the majority of structures found in the RNApedia database. Ribosomes are present in both prokaryotic and eukaryotic organisms and are responsible for the translational process. In eukaryotic organisms, two different ribosomes are generally found: the cytoplasmic ribosome, responsible for the synthesis of most proteins, and the mitochondrial ribosome (mitoribosome), responsible for the synthesis of a few proteins within the respiratory complex (Wang et al., 2024). While cytoplasmic ribosomes are highly conserved, mitochondrial ribosomes exhibit substantial structural divergence across species and are formed by a deep network of protein-RNA interactions. However, in some cases, this network of interactions includes peculiar components, with proteins partially replacing structural roles traditionally fulfilled by rRNA. This is the case of *Toxoplasma gondii* mitoribosome. This eukaryotic organism is a widespread parasite that infects a large fraction of the global population and can cause severe disease in immunocompromised individuals (Wang et al., 2024). In this use case, we will use the RNApedia database to explore the structure of the mitoribosome of *T. gondii.*

The mitochondrial ribosome of *T. gondii* is divided into two subunits: LSU (larger subunit) and SSU (smaller subunit). In contrast to most organisms, whose mitoribosomes consist of two or three long mt-rRNA chains, the mitoribosome of *T. gondii* is characterized by extensive rRNA fragmentation, featuring over 40 individual RNA strands integrated into its architecture (Wang et al., 2024). Interestingly, large mt-rRNA domains were lost during fragmentation throughout the organism’s evolution. This raises fundamental questions about how the mitoribosome remains functional despite the absence of multiple conserved mt-rRNA domains.

**FIGURE 4:**
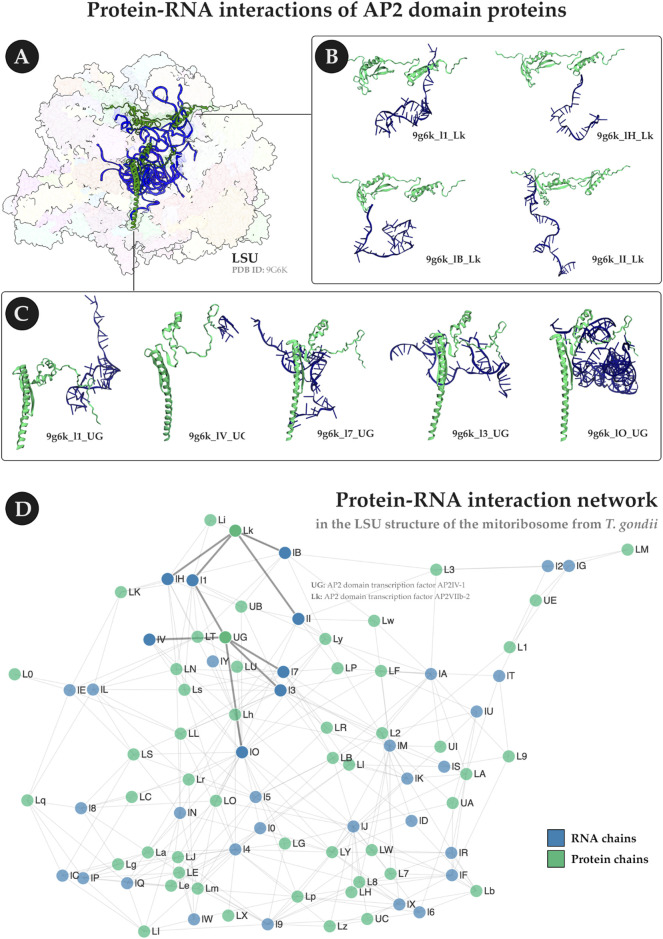
**(A)** Mitoribosome LSU structure (PDB ID: 9G6K). **(B)** Interactions among Lk chain and four RNA fragments (l1, lH, lB, and lL chains). **(C)** Interactions among UG chain and five RNA fragments (l1, lV, l7, l3, and lO chains). **(D)** Protein-RNA interaction graph. Labels represent the chain names. Blue spheres represent RNA fragments and green spheres are protein chains. Lines represent protein-RNA interactions.

When analyzing the *T. gondii* LSU structure (Figures 4A–C), we observed numerous proteins interacting with the RNA (Figure 4D). Thus, we hypothesized that proteins were assuming structural roles that belonged to those mt-rRNA domains. Hence, we selected the LSU structure (Figure 4A) of the mitochondrial ribosome of *T. gondii* (PDB ID: 9G6K). A search in RNApedia returned 284 protein-RNA interaction pairs for this complex. Then, we plotted a protein-RNA interaction network (Figure 4D). We highlight the AP2 proteins, which in the mitoribosome of *T. gondii* appear to be crucial for stabilizing this molecular machine.

In the 9G6K complex, the AP2 domain is present in two proteins (Lk and UG chains) that are essential for translation in this organism. The Lk chain has two AP2 domains, while UG has only one. These two proteins do not interact with each other, but they do interact with the extremities of several RNA fragments. The Lk chain interacts with l1, which in turn interacts with UG (Figure 4D). RNApedia detected interactions of the Lk chain with four RNA fragments (Figure 2B). In addition, we detected five interactions with the UG chain (Figure 4C). This suggests that these two proteins act as RNA links, playing a crucial role in stabilizing the complex by filling the gaps left by the absent mt-rRNA domains. Also, this suggests the AP2 protein-binding site is a promising target for the design of specific drugs against *T. gondii*.

**FIGURE 5:**
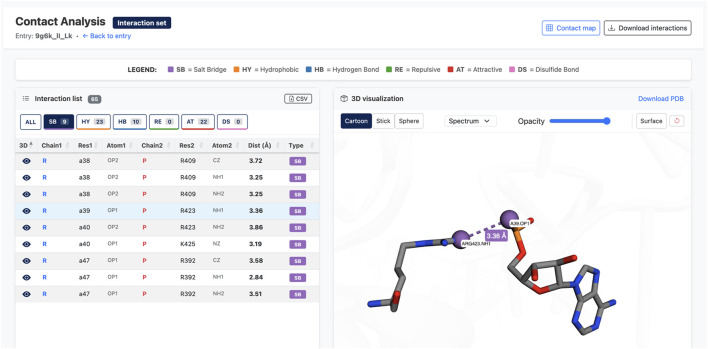
RNApedia contact analysis tool for strands lI and Lk of PDB 9G6K. On the left, we see the list with the nine salt-bridge contacts. On the right, we see the stick contact between the OP1 atom of nucleotide A:39 and the NH1 atom of R423.

Consider the interactions between the lI and Lk chains (Figure 3B- bottom right). RNApedia detected an interaction interface of 413 Å^2^. Wang et al. (2024) report the importance of R423 for measuring interactions between AP2 (Lk strand) and RNA strands. The RNApedia contact analysis tool provides a level of refinement in interatomic interaction analysis, detecting 65 possible contacts at the lI and Lk contact interface (Figure 5). These include nine salt bridges, 23 hydrophobic interactions, 10 hydrogen bonds, and 22 attractive interactions. In the example in Figure 4 (right), we can see the salt bridge between adenine nucleotide (A39) and arginine (R143).

#### 3.2.2 Use case II: structural and functional characterization of the KSRP protein from Trypanosoma cruzi (PDB ID: 5OPT)

**FIGURE 6:**
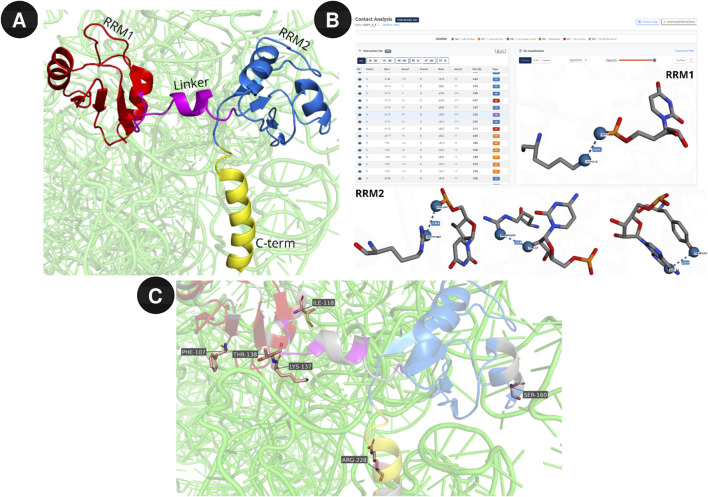
Structural and interaction features of KSRP from *Trypanosoma cruzi* (PDB ID: 5OPT). **(A)** Three-dimensional representation of the KSRP protein (chain h) highlighting its domain organization, including the RNA-recognition motifs (RRM1 and RRM2), the linker region, and the C-terminal segment. These domains are associated with RNA binding and structural stabilization. **(B)** Protein-RNA interaction interface of KSRP identified using the RNApedia. Interatomic contacts between protein and RNA atoms are shown, with emphasis on hydrogen bonds, which constitute the predominant interaction type at the interface. The interactions are mainly localized within the RRM domains. **(C)** Surface representation of KSRP highlighting regions with high contact density and solvent accessibility as identified by RNApedia.

To demonstrate the relevance of RNApedia in the structural and functional analysis of RNA-binding proteins, we have studied the kinetoplastid-specific ribosomal protein (KSRP) from *Trypanosoma cruzi* (PDB ID: 5OPT) as a case report. Within the kinetoplastid ribosome, KSRP is associated with the 40S subunit, where it helps maintain the organization of specific regions of ribosomal RNA (rRNA). Because no homolog has been identified in host organisms and its architecture differs from canonical ribosomal proteins, KSRP stands out as a potential entry point for the development of selective antiparasitic strategies (Querido et al., 2017). To examine whether the regions detected by RNApedia are particularly relevant, we conducted a separate computational analysis using the same protein structure for comparison. Approaches such as molecular docking, inspection of interatomic contacts, solvent accessibility, and residue flexibility were applied to identify regions of the protein that may contribute to KSRP function. Structurally, KSRP comprises RNA recognition motifs (RRMs), including two main domains (RRM1 and RRM2), linked by a flexible linker region, and a C-terminal segment. These domains are known to mediate RNA binding and are expected to play a central role in interface formation, which represents the protein’s three-dimensional structure. Figure 6A exhibits the domain organization.

The KSRP-RNA interaction was evaluated using the same process applied to the RNApedia dataset to ensure methodological coherence. Protein-RNA pairs were defined using an interatomic distance cutoff of ≤6.0 Å, enabling the identification of interactions important for molecular recognition. Interatomic contacts were then computed according to their physicochemical properties, including hydrogen bonds, hydrophobic interactions, and aromatic contacts. The contact analysis showed that hydrogen bonds are the most frequent interactions at the protein-RNA interface, in agreement with RNApedia.

Interface properties were evaluated using solvent-accessibility and surface-area parameters, including Accessible Surface Area (ASA), ΔASA, and Buried Surface Area (BSA), as described in the RNApedia workflow. Regions with higher ΔASA values are mainly located in exposed segments of the RNA-recognition motifs, indicating their direct involvement in RNA binding. The residues K115 (RRM1) and R177, Y181, and R182 (RRM2) establish multiple contacts with RNA nucleotides, forming interaction clusters (Querido et al., 2017). These include hydrogen bonds and salt bridges with phosphate groups, as well as hydrophobic contacts involving aromatic residues, particularly Y181 interacting with nucleotide bases (Querido et al., 2017). This behavior reflects a continuous, binding interface in which multiple residues simultaneously involve RNA nucleotides. The spatial distribution of these interaction regions is shown in Figures 6B,C.

The different computational strategies converge, suggesting that the interaction patterns in RNApedia reflect the structural and functional features of the protein-RNA interface. It is considered complementary evidence supporting the consistency of RNApedia descriptors in identifying contact hotspots. In a biological context, KSRP is positioned on the solvent-accessible surface of the 40S ribosomal subunit, is associated with expanded rRNA regions, and contributes to the stabilization of structural elements. The evaluated regions align with this role and are biologically consistent with the experimental information from the literature. In general, this case study demonstrates that RNApedia may predict key structural and interaction features of RNA-binding proteins, including contacts, interface properties, and functionally relevant regions. Existing agreement with independent computational approaches, together with its consistency with established structural and biochemical fundamentals, reinforces RNApedia’s potential as a reliable resource for large-scale analyses and targeted investigations. Finally, the identification of interaction hotspots and structurally relevant regions suggests that RNApedia is a useful platform for structure-based drug design targeting RNA-binding proteins (Querido et al., 2017).

### 3.3 Comparison to other tools

In this section, we compare the resources provided by RNApedia with those of other databases that provide protein-RNA complexes from the literature. Assessing the current landscape of databases storing structural information on protein-RNA complexes, we observe a wide variety. However, it is considerably fragmented, reflecting the specialization of each resource in specific aspects of RNA biology and molecular interactions, which hinders the search for this data *in silico* analyses. As summarized in Table 2, the available databases differ substantially in scope, types of information, availability, and degree of annotation.

**TABLE 2:** Overview and comparison of databases containing protein-RNA structures.

Database	RNApedia	PRIDB	RsiteDB	NPIDB	RNAproDB	ProNAB	PDBbind
Entries	56,133	926	2,300	5,000	3,500	5,326	1,584
Protein-RNA specific	✔	✔	✔	✖	✖	✖	✖
Update status	Apr 2026 (quarterly)	unavailable	unavailable	unavailable	PDB synchronized	Feb 2023	Public release: 2020
Atomic contacts	✔	✔	✔	✔	✔	✖	✖
Interface area (BSA/ASA)	✔	✖	✔	✔	✔	✖	✖
RNA structure annotation	✔	✖	✖	✔	✔	✖	✖
Protein domains (Pfam)	✔	✔	✔	✔	✖	✖	✖
RNA modifications	✔	✖	✖	✖	✖	✔	✖
Binding affinity	✔	✖	✖	✖	✖	✔	✔
Structured downloadable dataset	✔	✖	✖	✖	✖	✔	✔
Web visualization available	✔	✖	✖	✖	✔	✖	✖

The reported release year corresponds to the most recent publicly accessible version available at the time of manuscript preparation.

Several databases have been developed to support the study of protein–RNA interactions, reflecting a heterogeneous and fragmented landscape of resources. Among the databases summarized in Table 2, PRIDB (Protein–RNA Interface Database) (Lewis et al., 2011) and RsiteDB (Shulman-Peleg et al., 2009) provide curated information on protein–RNA interaction sites and structural motifs. However, these resources are more limited in terms of data coverage, update status, and integration of multiple annotation layers, which restricts their applicability to large-scale computational analyses.

More comprehensive resources, such as RNAproDB (Mitra et al., 2025), provide relevant structural information on protein–RNA interactions and are systematically updated. Nevertheless, RNAproDB differs from RNApedia in scope and annotation strategy, as it does not integrate the same combination of experimental affinity annotations, protein domain information, RNA modifications, pair-level interface descriptors, and structured downloadable datasets within a single resource.

NPIDB (Nucleic Acid–Protein Interaction Database) (Kirsanov et al., 2013) provides structural data on protein–nucleic acid complexes, including protein–RNA interactions, and includes annotations of intermolecular contacts. However, it is not specifically focused on protein–RNA systems, as it covers a broader range of nucleic acid–protein complexes. In addition, its applicability to current large-scale computational workflows is limited by its maintenance status and by the lack of integration with experimental affinity data and RNA-specific annotations.

Databases such as PDBbind (Wang et al., 2005; Wang et al., 2004) and ProNAB (Harini et al., 2022) provide valuable experimental binding affinity data, including dissociation constants and thermodynamic parameters. Despite their relevance for affinity-related analyses, these resources primarily focus on binding affinity information and do not provide the same level of integrated structural characterization of protein–RNA interfaces, such as atomic contact classification, interface area descriptors, RNA secondary-structure annotations, protein domain information, and RNA modification mapping.

In contrast, RNApedia adopts an integrative approach that consolidates multiple layers of structural and functional information into a single resource. Beyond structural data, RNA and protein classification, protein domain annotation, and detailed interface characterization, including atomic contacts and interface area descriptors, RNApedia provides structured downloadable datasets and an accessible web-based visualization platform. Together, these features support large-scale computational analyses and interactive data exploration. RNApedia is also designed as a continuously maintained resource, with planned quarterly updates incorporating newly released protein–RNA structures and additional annotations.

### 3.4 Limitations

Although RNApedia has broad potential for structural and computational analyses of protein–RNA interactions, some limitations should be considered. First, RNApedia inherits the intrinsic biases of the PDB, which is heavily enriched in ribosomal complexes. This bias is further amplified by the pair-level organization adopted in RNApedia, since large ribosomal assemblies contain multiple protein and RNA chains and can therefore generate a large number of protein–RNA interaction pairs from a single structure. This overrepresentation reflects both the biological relevance and the intense scientific interest in ribosomal complexes, as well as recent advances in cryo-electron microscopy that have enabled the structural determination of large macromolecular assemblies. In contrast, several classes of non-ribosomal RNA, particularly small regulatory RNAs, long non-coding RNAs, and viral RNAs, remain underrepresented due to their conformational flexibility, transient binding modes, and experimental challenges associated with structural determination. To facilitate analyses using more balanced subsets, RNApedia provides RNA-type annotations and downloadable rRNA and non-rRNA datasets, enabling users to stratify analyses by the biological question being addressed. This is particularly important for computational analyses and machine learning applications, where the imbalance in the representation of rRNA-containing complexes can bias model training, evaluation, and interpretation.

A second limitation is the restricted coverage of experimentally measured binding affinity data, which are available for only a small fraction of the entries. This limitation reflects the scarcity of publicly available experimental affinity data for protein–RNA complexes and is particularly relevant because protein–RNA affinity prediction remains an active and challenging research area. Previous studies have shown that experimentally measured protein–RNA affinities are limited and strongly dependent on experimental conditions, including temperature, pH, ionic strength, and the measurement method used (Yang et al., 2013; Harini et al., 2024; Krautwurst and Lamkiewicz, 2024; Nithin et al., 2019). Therefore, applications that rely on quantitative interaction measures, such as affinity prediction, should account for this limited coverage. The structural descriptors available in RNApedia, including buried surface area, interface area, contact density, hydrogen bonds, interaction composition, and contact-type profiles, may be useful as explanatory features in computational models. Previous structure-based affinity prediction studies have used interface descriptors, conformational changes, hydration patterns, atomic contacts, hydrogen bonds, interaction energies, contact potentials, and RNA structural parameters to model protein–RNA binding affinity (Yang et al., 2013; Harini et al., 2024; Krautwurst and Lamkiewicz, 2024). However, these descriptors should not be interpreted as direct substitutes for experimentally measured affinities without further validation. Instead, they should be considered structural and physicochemical features that may support future affinity-prediction workflows when combined with experimentally validated Kd or ΔG values. Accordingly, RNApedia provides a downloadable affinity subset to facilitate analyses restricted to entries with available experimental binding information.

The comparison with existing resources indicates that protein–RNA databases differ in coverage, annotation depth, update status, and usability in computational workflows. RNApedia contributes to this landscape by integrating pair-level organization, structural descriptors, atomic contact classification, interface area measurements, RNA structural annotations, protein domain information, RNA modifications, downloadable datasets, and web-based visualization in a single resource.

Nevertheless, broader external validation remains an important future direction. Future versions may include quantitative comparisons with independent protein–RNA interaction datasets, affinity benchmarks, docking benchmarks, or curated structural benchmarks. Such analyses would help evaluate the consistency of RNApedia annotations, the generalizability of its descriptors across different biological contexts, and their applicability in downstream computational workflows.

RNApedia also relies on experimentally resolved static structures, which represent specific conformational states captured under particular experimental conditions. Protein–RNA interactions are dynamic and may involve transient contacts, multiple conformational states, and binding-induced rearrangements that are not fully captured by deposited structures. Therefore, RNApedia should be interpreted as a resource describing experimentally observed structural states rather than the full conformational landscape of protein–RNA recognition. Future versions may incorporate molecular dynamics simulations, conformational ensembles, multiple structural states, or predicted models generated by recent artificial intelligence-based methods to better represent the structural plasticity of protein–RNA interactions.

Finally, RNApedia organizes entries as protein–RNA pairs, each composed of one protein and one RNA chain. This pair-level organization enables detailed interface-level analyses, particularly in large multichain complexes, by allowing individual protein–RNA interfaces to be examined separately. However, it does not directly represent the full architecture of the original macromolecular assembly. Therefore, RNApedia entries should be interpreted as interaction-level units rather than complete complex-level representations. Users interested in the global organization of multichain assemblies should complement RNApedia pair-level information with the corresponding original PDB structures.

## 4 Conclusion

RNApedia is a comprehensive and curated database designed to aid in the structural analysis of protein-RNA complexes through an accessible and intuitive framework. Its pair-based organization allows for detailed characterization of interaction interfaces, even in large macromolecular assemblies. Analyses performed with RNApedia reveal that protein-RNA interfaces are highly heterogeneous and multifactorial, predominantly governed by electrostatic and hydrogen-bond interactions, with additional contributions from hydrophobic and aromatic interactions. The applicability of the database is demonstrated through case studies, including the mitoribosome of *Toxoplasma gondii,* where RNApedia enabled the identification of extensive interaction networks, and the use case of the KSRP protein from *Trypanosoma cruzi*, where it successfully identified functional regions and critical interaction points consistent with independent computational and experimental data. As protein–RNA interactions continue to gain relevance in molecular biology, biotechnology, and RNA-based therapeutics, RNApedia may serve as a useful platform for large-scale structural analyses and data-driven approaches, including potential applications in artificial intelligence. Future developments could focus on expanding the coverage of experimental affinity data, incorporating additional RNA classification strategies, integrating dynamic information, and maintaining regular updates in line with advances in the field.

## Data Availability

RNApedia is publicly available through a web interface at https://bioinfo.dcc.ufmg.br/rnapedia. All datasets, including curated protein-RNA pairs, structural annotations, and derived features, can be accessed, browsed, and downloaded without restriction. The database is also available at https://zenodo.org/records/20073649.
