# Supplementary material for: RNApedia: a database of structural protein–RNA interactions
Source: Front Bioinform. 2026 Jun 17;6:1857218. doi: 10.3389/fbinf.2026.1857218 (PMC13319083; doi:10.3389/fbinf.2026.1857218)
Supplement: Supplementary file 1 [file DataSheet1.pdf]

Supplementary Material

Table S1. Crystallographic artifacts

Abbreviation	Name
ACE	*Acetyl Group'*
ACT	*Acetate Ion*
BME	*Beta-mercaptoethanol*
EDO	*1,2-ethanediol*
FMT	*Formic Acid*
GOL	*Glycerol*
MSE	*Selenomethionine*
NAG	*N-acetyl-d-glucosamine*
NO_3	*Nitrate Ion*
PO_4	*Phosphate Ion'*
SGM	*Monothioglycerol*
$SO_{4'}$	*Sulfate Ion*
TPO	*Phosphothreonine*

Table S2. RNA modifications included in RNApedia.

Modification id	Modification name
6MA / M6A / 12A / 6MZ	N6-Methyladenosine
M6AM	N6,2′-O-Dimethyladenosine
2MA / M2A / A2M	2-Methyladenosine
1MA / M1A	1-Methyladenosine
M2M	2,2-Dimethyladenosine
M7A	7-Methyladenosine
MIA / I6A	N6-Isopentenyladenosine
A23	2,3-Dideoxyadenosine
A2G	2-Guanidinoadenosine
A3P	3′-Deoxyadenosine-3′-phosphate
BAU	Benzoyladenosine
T6A / TLA	N6-Threonylcarbamoyladenosine
TNM	2-Thio-N6-threonylcarbamoyladenosine
AET	2-Aminoethylthioadenosine
2OMA	2′-O-Methyladenosine
MA6	N6-Methyladenosine-5′-monophosphate
MGT	2-N-Methylguanosine-5′-triphosphate
N6G	N6-Glycinocarbamoyladenosine
23G	2,3-Dihydroguanosine
1MG / M1G	1-Methylguanosine
2MG / M2G / G2M	N2-Methylguanosine
7MG / M7G / G7M	7-Methylguanosine
M7M	7-Methyl-2′-O-methylguanosine
OMG / 2OMG	2′-O-Methylguanosine
QSI / Q	Queuosine
QUO	Queuine
GLZ	Glutamylqueuosine
Y	Wyosine
YG / YYG	Wybutosine (derivative)
YMP	Wybutosine monophosphate
M5C / 5MC	5-Methylcytidine
M3C	3-Methylcytidine
M4C	4-Methylcytidine
AC4C	N4-Acetylcytidine
OMC / 2OMC	2′-O-Methylcytidine
5HC	5-Hydroxycytidine
5IC	5-Iodocytidine
5CY	5-Carboxycytidine
A2U / S2U	2-Thiouridine
2MU	2-Methyluridine
5MU / M5U	5-Methyluridine

PSU / Ψ	Pseudouridine
H2U / D	5,6-Dihydrouridine
4SU / S4U	4-Thiouridine
OMU / CHM	2′-O-Methyluridine
U34	5-Carbamoylmethyluridine
U37	2-Thiouridine (position 37)
U3H / 5HO	5-Hydroxyuridine
U5P	5-Phosphouridine
U6A	6-Azauridine
U7V	7-Methyl-2′-O-methyluridine
U7Y	7-Methyl-2-thiouridine
M1Y	1-Methylpseudouridine
MNM5U / MRC	5-Methylaminomethyluridine
MNM5GES2U	5-Methylaminomethyl-2-geranylthiouridine
MCM5U	5-Methoxycarbonylmethyluridine
MCM5S2U	5-Methoxycarbonylmethyl-2-thiouridine
TTE	5-Taurinomethyluridine
GEA	Geranyl-2-thiouridine
CBV	Carboxyvinyluridine
PRT	2-Propenyluridine
5FU	5-Fluorouridine
5BU / BRU	5-Bromouridine
CBR	5-Carboxyuridine
5OD	5-Oxo-2-thiouridine
TSB	2-Thiouridine-5′-triphosphate
UCR	5-Carboxymethylaminomethyluridine
UMS	Ribothymidine (m5U monophosphate)
UR3	Uridine-5′-monophosphate-3′-uridine

Table S3. Database composition according to RNA type.

RNA_type	protein-RNA pairs	percentage %	PDBs unique IDs
rRNA	38,068	67.8	1,248
misc_RNA	3,861	6.9	1,173
Synthetic RNA	3,834	6.8	1,156
snRNA	2,459	4.4	133
tRNA	2,423	4.3	712
Unclassified	2,086	3.7	854
mRNA	1,153	2.1	294
guide_RNA	526	0.9	215
ncRNA	208	0.4	67
IRES	175	0.3	27
vRNA	167	0.3	54
srRNA	116	0.2	9
Target RNA	82	0.1	20
telomerase_RNA	80	0.1	21
DGR RNA	78	0.1	9
SRP_RNA	73	0.1	23
sRNA	65	0.1	28
snoRNA	47	0.1	10
Retron RNA	43	0.1	6
Genomic Transcript	43	0.1	1
Template RNA	39	0.1	15
Nascent RNA	38	0.1	7
ribozyme	35	0.1	19
RNase_P_RNA	34	0.1	10
RNase_MRP_RNA	33	0.1	5
Product RNA	31	0.1	12
siRNA	31	0.1	11
tmRNA	30	0.1	5
ITS2 RNA	27	0	10
rut RNA	24	0	7
Primer RNA	23	0	18
Cyclic Oligonucleotide	22	0	6
miRNA	19	0	9
riboswitch	16	0	9
Lariat Intron	16	0	3
Y RNA	14	0	4

Aptamer	13	0	8
eRNA	11	0	3
Cyclic RNA	10	0	5
UTR	10	0	5
PolyA RNA	8	0	2
omegaRNA	8	0	4
Capped RNA	8	0	4
ssRNA	8	0	5
7SK RNA	6	0	4
Precursor RNA	6	0	3
piRNA	5	0	5
cis-regulatory RNA hairpin	4	0	2
Hybrid RNA/DNA	4	0	2
tRNA fragment	4	0	3
PolyU RNA	3	0	1
genomic ssRNA	2	0	1
lncRNA	2	0	1
nucleocapsid RNA	1	0	1
cis-regulatory RNA	1	0	1

Table S4. Comparative distribution of interaction types across protein–RNA, rRNA, non-rRNA and modified RNA interfaces

Interaction Type	Overall (%)	rRNA (%)	non-rRNA (%)	Modified RNA (%)
AT	36.16	17.80	36.00	22.30
HB	34.27	28.50	34.00	23.66
HY	15.02	52.70	12.00	10.20
SB	11.37	0.80	14.00	6.40
RE	3.18	0.20	4.00	37.93

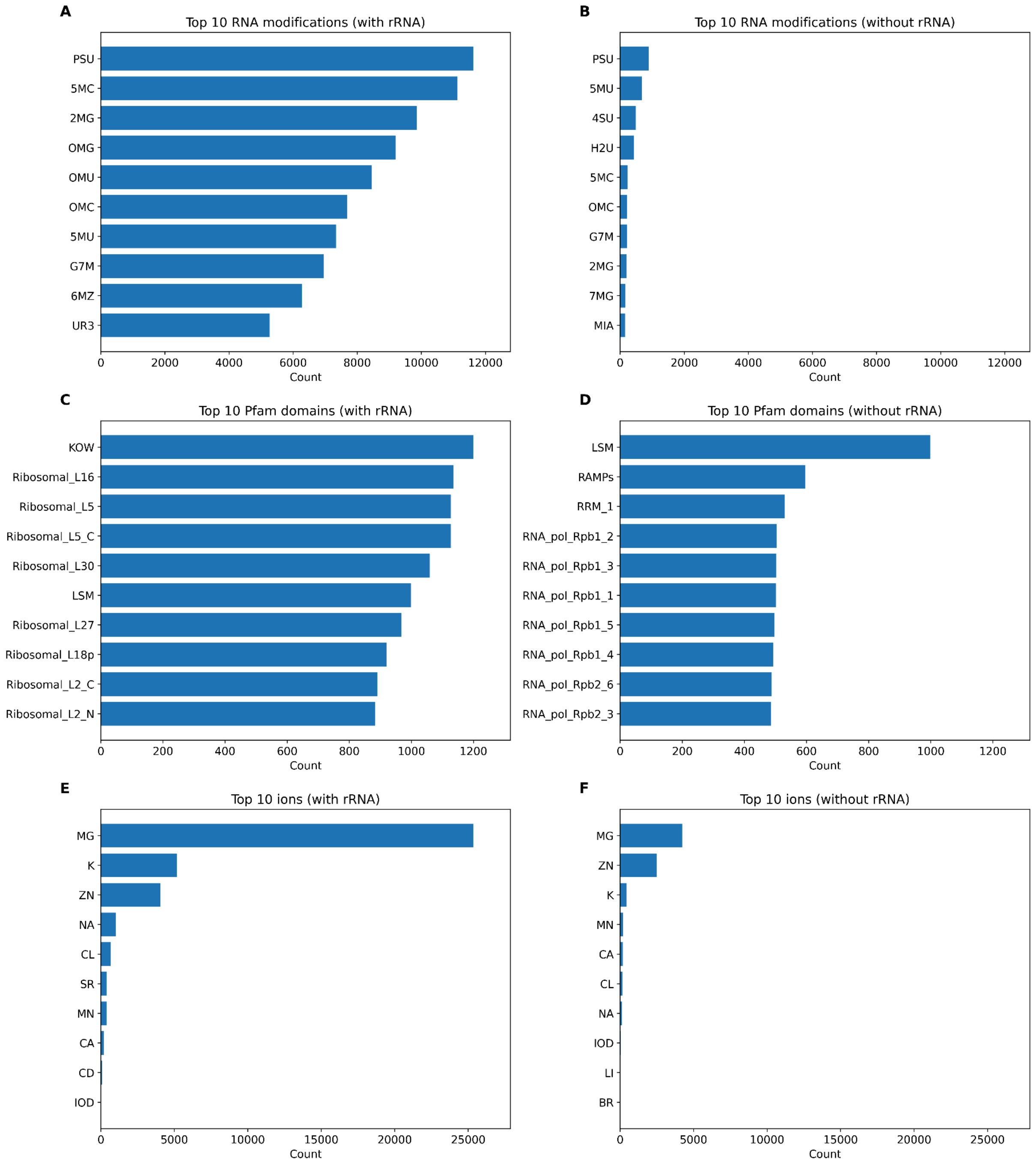

Figure S1. Top 10 RNA modifications (A–B), Pfam binding domains (C–D), and ions (E–F) in RNApedia. Panels A, C, and E correspond to the complete dataset, including ribosomal RNAs (rRNAs), while panels B, D, and F represent the dataset after excluding rRNA. For each category, the y-axis scale is kept constant between paired panels, allowing for direct comparison.

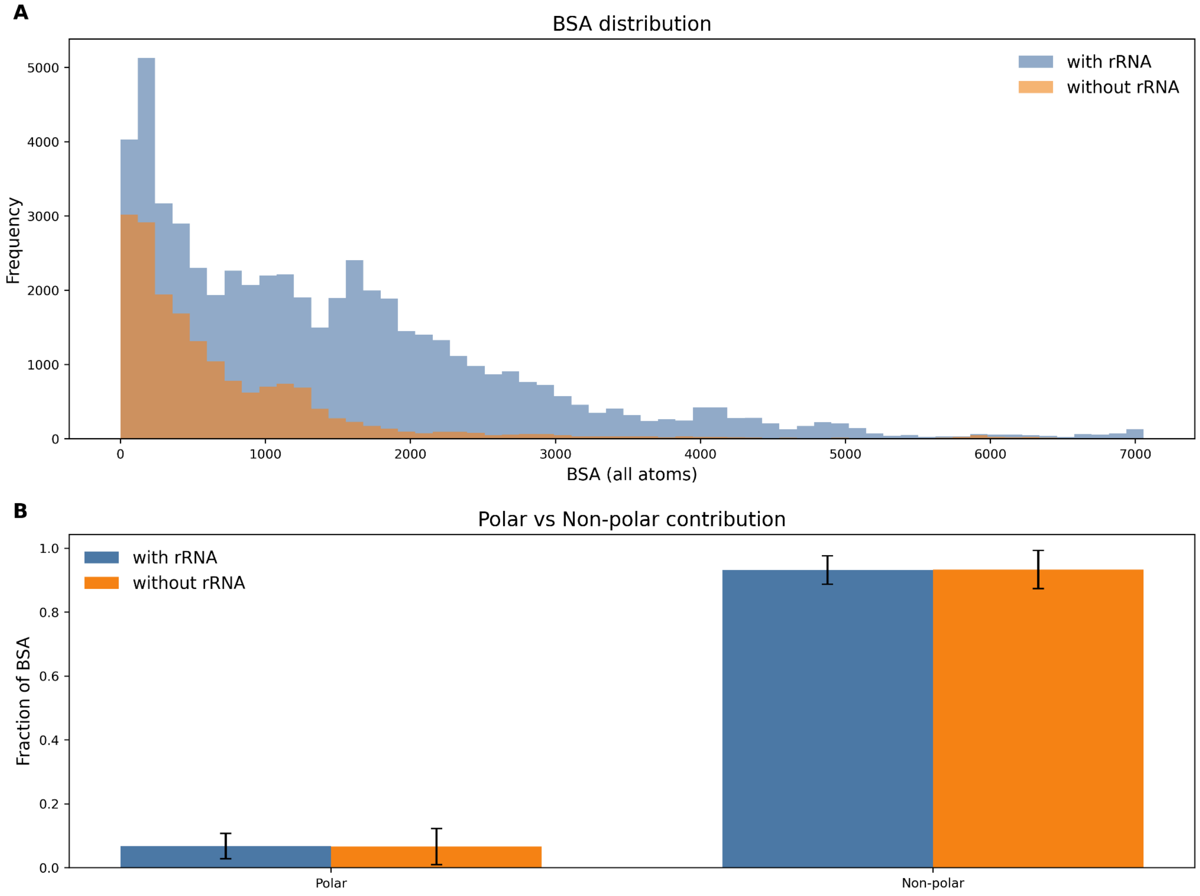

Figure S2. Distribution of buried surface area (BSA) in protein-RNA pairs present in RNApedia. (A) Distribution of total BSA in the complete set (with rRNA) and after removal of ribosomal RNAs (without rRNA). (B) Average fraction of polar and non-polar contributions in the two datasets.

Supplementary Text S.1

RNApedia is a comprehensive, curated database with a user-friendly web interface, designed to simplify navigation and searching for protein-RNA complex structures (Figure S3). The platform was conceived to support research in structural biology and bioinformatics, integrating structural, sequence, and molecular interaction information in a single, easily accessible environment.

In addition to facilitating the identification of specific targets, RNApedia enables the acquisition of large volumes of protein-RNA complex data, thereby facilitating the construction and training of artificial intelligence-based models. Therefore, the database has significant potential to contribute to the study, characterization, and understanding of protein-RNA interactions and their biological and structural properties.

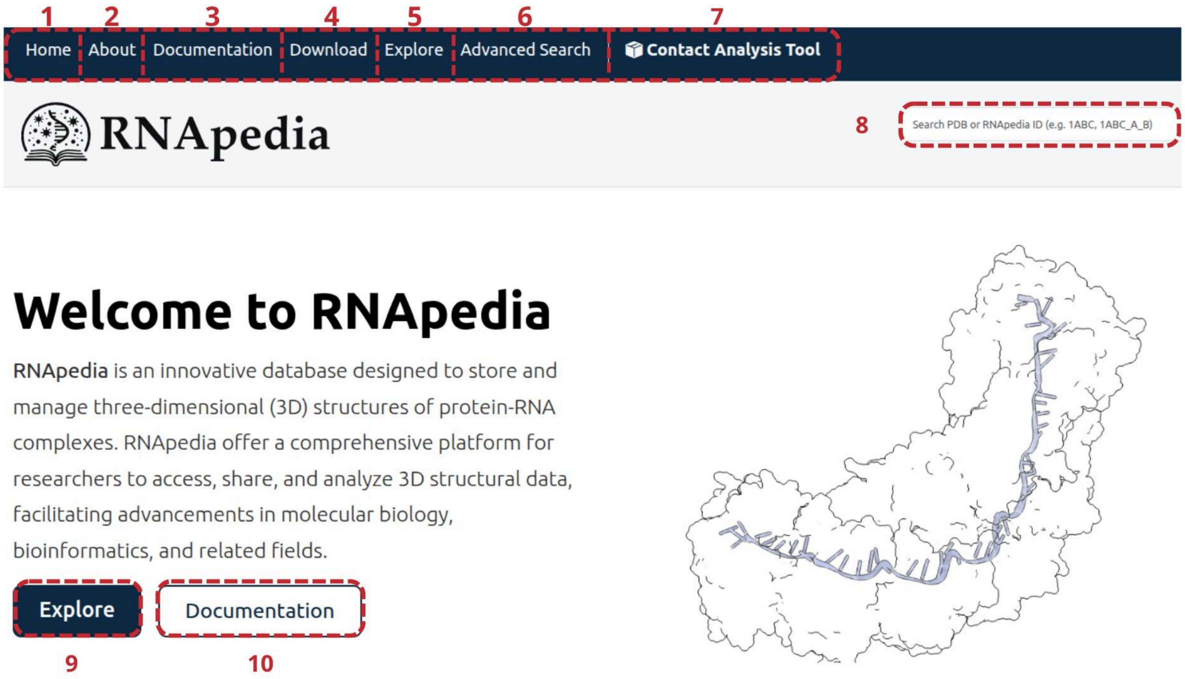

Figure S3. Initial RNApedia interface. (1) home page, (2) general project information, (3) detailed documentation, (4) downloads page, (5) database exploration, (6) advanced search with filters and structural functionals, and (7) a dedicated tool for analyzing protein-RNA contacts. The interface also includes (8) a quick search field for PDB identifiers or RNApedia IDs. In the central area, there are buttons for direct access to the main functionalities: (9) database exploration and (10) platform documentation.

On the RNApedia Download page (Figure S4), we provide different datasets organized in a clear and intuitive way. We offer the complete RNApedia dataset, as well as subsets formed

by complexes with available experimental affinity, the presence of modified nucleotides, and identified binding motifs in the protein structure. To facilitate sequence analysis, it is also possible to download multi-FASTA files of protein and RNA sequences. This organization facilitates both exploratory use and application in large-scale analyses, including the development of machine learning and artificial intelligence models focused on protein-RNA interactions.

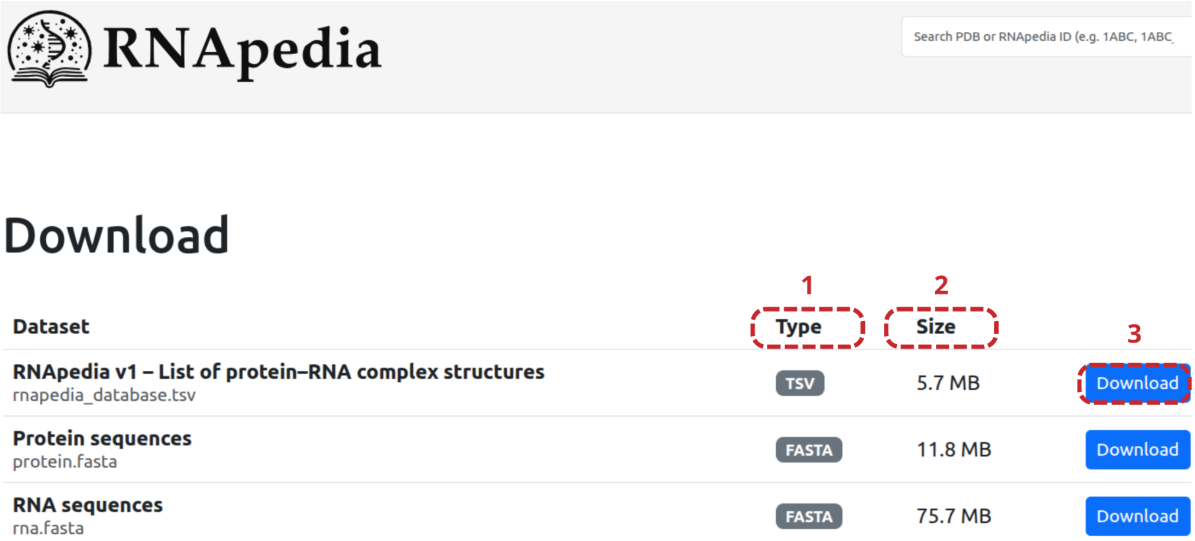

Figure S4. RNApedia Download Page. The interface displays the datasets available for download, indicating (1) the file type (TSV, ZIP, or FASTA), (2) the file size, and (3) the access button for direct download. The data range from summary lists of protein-RNA complexes to complete datasets containing structures, sequences, and functional annotations.

Each entry has a specific page (Figure S5). In this interface, the user can access a three-dimensional visualization of the complex, enabling spatial inspection of the protein and RNA together. In parallel, the platform presents a summary panel with experimental and structural metadata, including the method of structure determination, resolution, the number of protein and RNA chains, and information on ligands, ions, and global interface metrics.

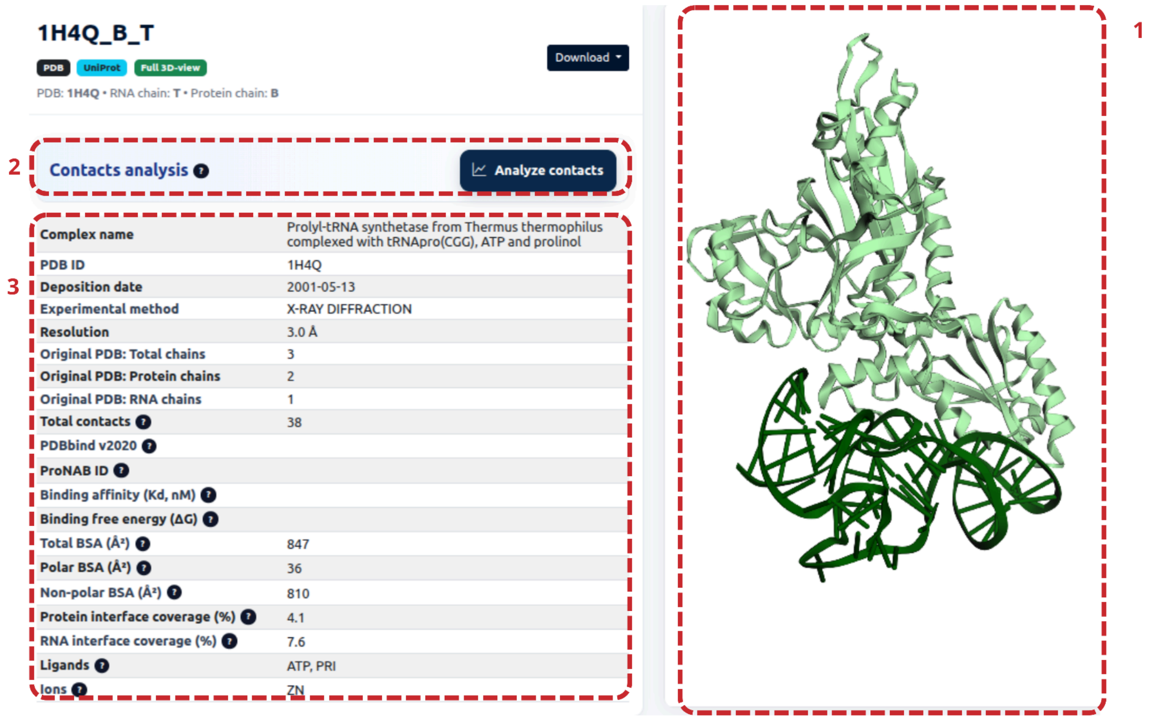

Figure S5. Overview of the analysis page for each entry of the protein-RNA complex in RNApedia. The interface presents structural and functional information of the 1H4Q_B_T complex, integrating experimental data, interaction metrics, and three-dimensional visualization. (1) 3D visualization of the protein-RNA complex, allowing spatial inspection of the interaction interface. (2) Contacts analysis section, where the user can start the detailed analysis of the intermolecular contacts. (3) Complex metadata panel, including PDB identification, experimental method, resolution, number of chains, total contacts, buried surface areas (BSA), interface coverage, presence of ligands and ions.

In the RNA section of each entry's page (Figure S6), you can access RNA chain data, including ID, organism, description, and RNA type classification. In addition, a clickable sequence visualization is provided, which can be viewed in the three-dimensional structure, as well as nucleotide modifications that can be visualized in the structure. It is also possible to visualize the secondary structure of the RNA molecule, calculated from the sequence (RNAfold)(18) and the structure (DSSR)(17).

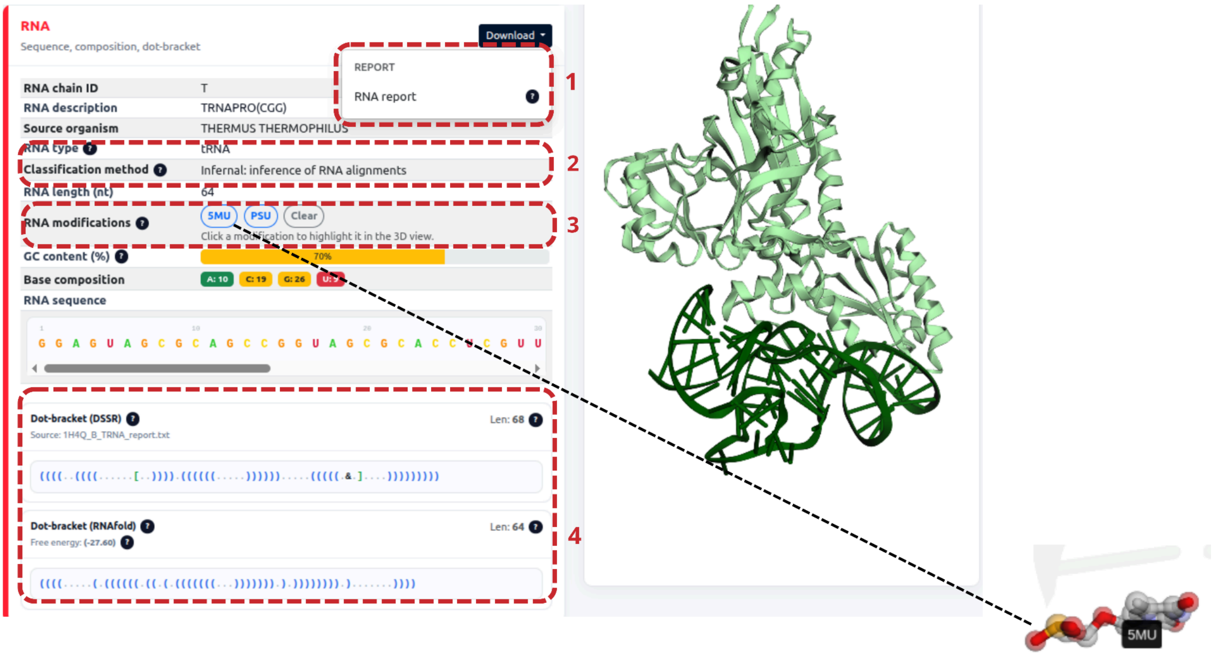

Figure S6. RNA structural information and analysis panel in RNApedia. The panel presents detailed information on the selected RNA chain, including chain identification, functional description, organism of origin, RNA type, and classification method. The RNA report option (1) allows downloading a complete report containing RNA annotations and analyses. The RNA type and Classification method section (2) informs the functional class of the RNA and the method used for its annotation, such as alignment inference (Infernal). The RNA modifications area (3) highlights the chemical modifications present in the sequence, which can be selected for visualization and direct highlighting in the three-dimensional structure. The lower Dot-bracket section (4) presents the secondary structure of the RNA in DSSR and RNAfold formats, allowing comparison between experimentally and computationally derived structures. On the right, the 3D visualization shows the protein-RNA complex, with the RNA highlighted, and the magnification reveals a specific modification mapped to the atomic structure.

In the protein section (Figure S7), in addition to organism data, description, and chain ID, properties such as isoelectric point, hydrophobicity, aromaticity, and stability are provided. Furthermore, as with RNA, the sequence is available and can be interactively visualized in 3D. Linkage motifs classified by Pfam are also provided when found in the sequence.

On the page, you can download the processed RNApedia structure, as well as the original PDB and fasta archive (29,30). Accessibility reports are also available, containing the NACCESS (29,30) calculated data for the complex and by residue. An RNA report, containing RNA pairing and stability parameters, and finally a protein report containing identification of secondary protein structures, calculated with STRIDE (19) , as well as functional domains identified using Pfam.

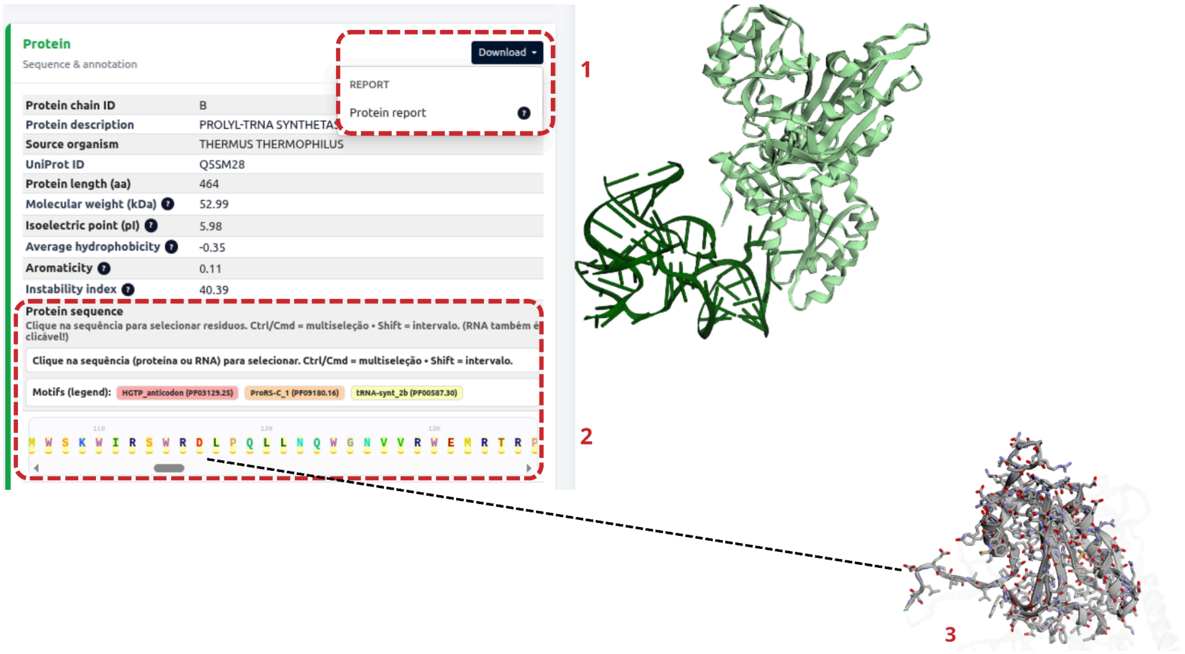

Figure S7. Information panel, functional annotation, and structural visualization of the protein in RNApedia. The panel presents detailed information on the selected protein chain, including chain identification, functional description, organism of origin, and UniProt identifier as well as overall physicochemical properties such as sequence length, molecular mass, isoelectric point, average hydrophobicity, aromaticity, and instability index. The Protein report option (1) allows the download of a complete report containing the structural and functional annotations of the protein. The lower section Protein sequence (2) displays the amino acid sequence interactively, allowing the selection of individual or multiple residues directly in the sequence, with support for range selection and multiselection, automatically reflecting the chosen regions in the three-dimensional visualization. The binding motifs (3) identified by Pfam domains are highlighted along the sequence. They can be correlated with their spatial locations within the 3D structure, facilitating analysis of the protein-RNA interface and the functional regions involved in molecular recognition.

Finally, each entry page has a Contacts analysis section that links to a new page containing the computational analysis of the interactions (Figure S8). In this section, contacts can be viewed in a downloadable table, a 3D visualization, and contact maps.

This integrated organization allows the user to progressively understand, from the general characteristics of the complex to the specific details of the interaction interface. By bringing together three-dimensional visualization, experimental metadata, and analytical tools in a single environment, RNApedia facilitates both exploratory analyses and large-scale systematic studies, advancing the structural understanding of protein-RNA interactions and the development of advanced computational approaches.

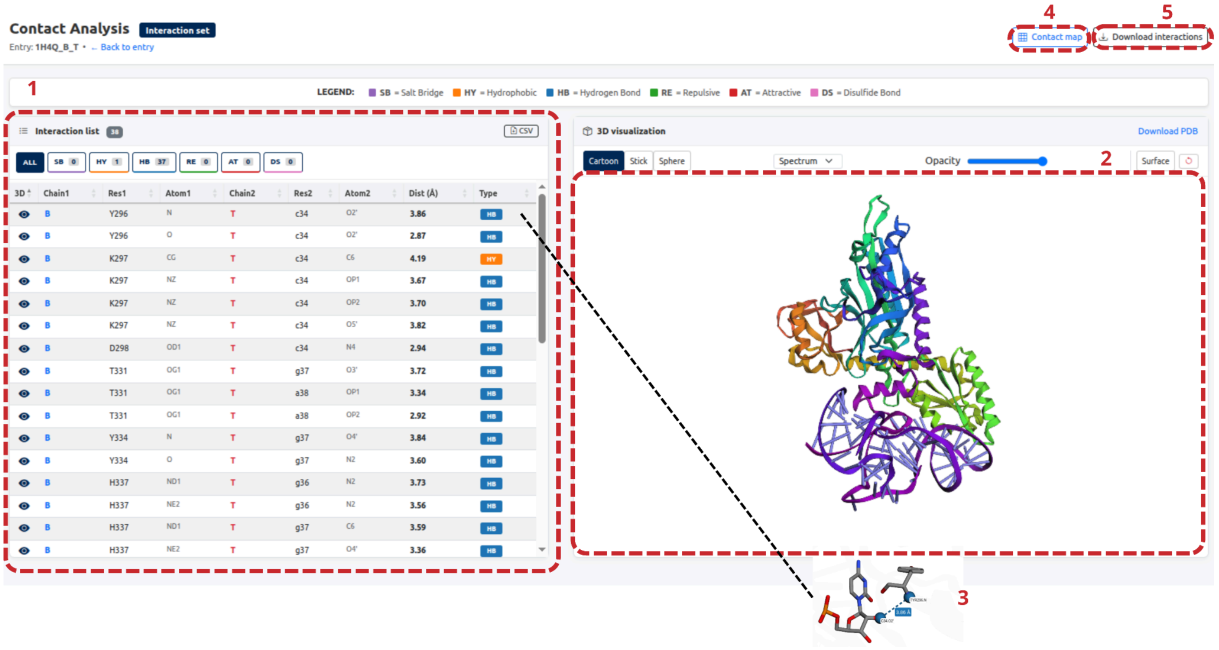

Figure S8. RNA-protein contact analysis tool in RNApedia. The Contact Analysis tool interface displays, on the left, a list of identified interactions (1) between protein and RNA atoms, including detailed information on the chains involved, residues, atoms, interatomic distance, and the type of interaction, classified as hydrogen bonding, hydrophobic interaction, salt bridge, attractive or repulsive interaction. On the right, the three-dimensional visualization (2) displays the protein-RNA complex, allowing switching between different representation styles (cartoon, sticks, and spheres), opacity and color control, as well as spatial inspection of the selected interactions. Selecting a specific interaction in the table automatically highlights the corresponding atomic contact (3) in the 3D visualization, facilitating detailed analysis of the interaction geometry. The Contact map button (4) allows access to a two-dimensional map of the interactions along the sequences, while the Download interactions option (5) allows downloading the contact data in tabular format for further analysis.

However, RNApedia has a page where it is possible to browse the data by database ID, structure resolution, RNA type, and number of contacts (Figure S9). It is also possible to perform an advanced and specific search using the RNA or protein sequence, as well as the organism, RNA type, type of modification present in the nucleotide, or linkage motif identified in the protein structure (Figure S10). Finally, the interface includes a tool for the user to calculate contacts for their protein-RNA structure of interest, with an interactive visualization page for data analysis (Figure S11)

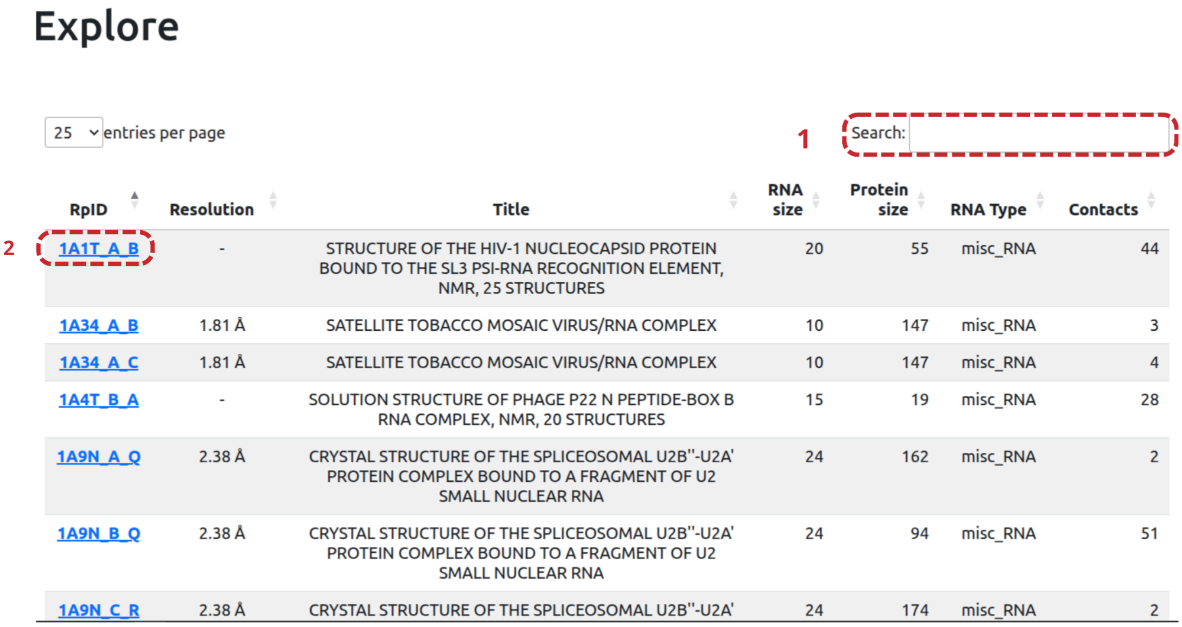

RpID	Resolution	Title	RNA size	Protein size	RNA Type	Contacts
1A1T_A_B	-	STRUCTURE OF THE HIV-1 NUCLEOCAPSID PROTEIN BOUND TO THE SL3 PSI-RNA RECOGNITION ELEMENT, NMR, 25 STRUCTURES	20	55	misc_RNA	44
1A34_A_B	1.81 Å	SATELLITE TOBACCO MOSAIC VIRUS/RNA COMPLEX	10	147	misc_RNA	3
1A34_A_C	1.81 Å	SATELLITE TOBACCO MOSAIC VIRUS/RNA COMPLEX	10	147	misc_RNA	4
1A4T_B_A	-	SOLUTION STRUCTURE OF PHAGE P22 N PEPTIDE-BOX B RNA COMPLEX, NMR, 20 STRUCTURES	15	19	misc_RNA	28
1A9N_A_Q	2.38 Å	CRYSTAL STRUCTURE OF THE SPLICEOSOMAL U2B''-U2A' PROTEIN COMPLEX BOUND TO A FRAGMENT OF U2 SMALL NUCLEAR RNA	24	162	misc_RNA	2
1A9N_B_Q	2.38 Å	CRYSTAL STRUCTURE OF THE SPLICEOSOMAL U2B''-U2A' PROTEIN COMPLEX BOUND TO A FRAGMENT OF U2 SMALL NUCLEAR RNA	24	94	misc_RNA	51
1A9N_C_R	2.38 Å	CRYSTAL STRUCTURE OF THE SPLICEOSOMAL U2B''-U2A'	24	174	misc_RNA	2

Figure S9. Interface of the RNApedia interactive table for exploring protein-RNA complexes. (1) Text search field that allows dynamically filtering the entries displayed in the table. (2) RNApedia identifier (RpID), which provides direct access to the individual page of the complex. The table presents, for each complex, structural and functional information, including experimental resolution, experiment title, RNA size, protein size, RNA type, and total number of molecular contacts.

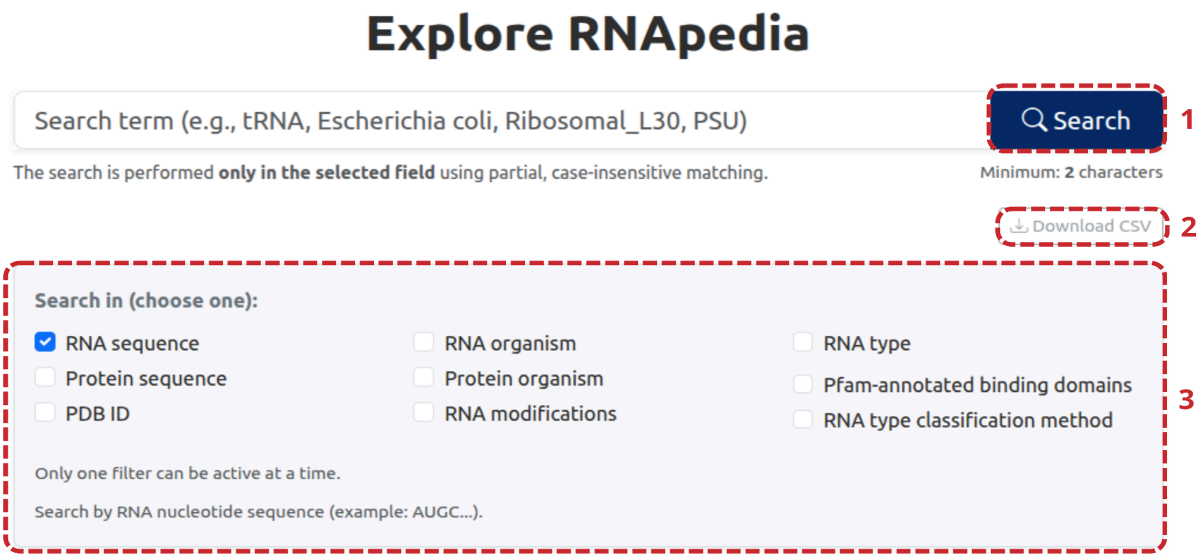

Figure S10. RNApedia advanced search interface for exploring protein-RNA complexes. (1) Search execution button after defining the term and field of interest. (2) Option to export the returned results in CSV format. (3) Search field selection panel, allowing specific queries by RNA sequence, protein sequence, organism, RNA type, PDB identifier, RNA modifications, Pfam-annotated binding domains, or RNA type classification method. Only one filter can be applied at a time, using partial matching and not case-sensitive.

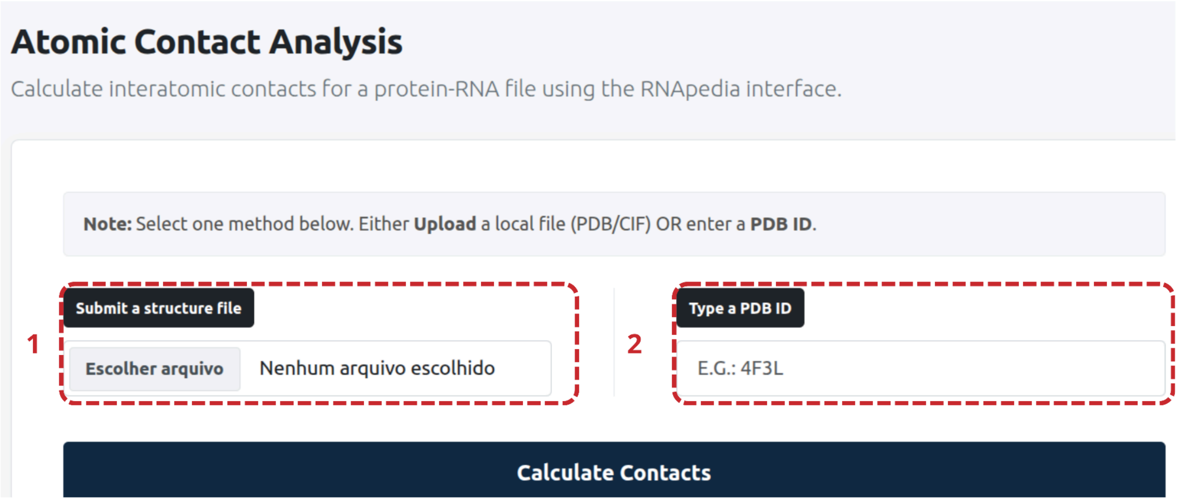

Figure S11. Interface of the RNApedia Atomic Contact Analysis tool for calculating interatomic contacts in protein-RNA complexes. (1) Submission of a local structural file in PDB or mmCIF format. (2) Alternatively, direct insertion of a PDB identifier for automatic structure retrieval. After submission, the tool calculates and returns the atomic contacts between protein and RNA using the criteria implemented in the RNApedia pipeline.

The web interface of RNApedia was developed with the main objective of facilitating access, exploration, and use of structural data on protein-RNA complexes by the scientific community. Therefore, the interface includes detailed documentation and help buttons to assist users. Navigation is intuitive, and the interface allows searching by structure, sequences, RNA types, protein domains, nucleotide modifications, and provides complete data for download and individual analysis. Thus, it is expected that the web interface will help broaden data accessibility and establish a robust environment for exploratory and large-scale analyses of protein-RNA interactions.
